# Dynein-driven regulation of postsynaptic membrane architecture and synaptic function

**DOI:** 10.1242/jcs.263844

**Published:** 2025-03-12

**Authors:** Amanda L. Neisch, Thomas Pengo, Adam W. Avery, Min-Gang Li, Thomas S. Hays

**Affiliations:** ^1^ Department of Genetics, Cell Biology, and Development, University of Minnesota, Minneapolis, MN 55455, USA; ^2^ University of Minnesota Informatics Institute, University of Minnesota, Minneapolis, MN 55455, USA

**Keywords:** Dynein, Glutamatergic neurons, Postsynaptic architecture, Neuromuscular junction

## Abstract

Cytoplasmic dynein is essential in motor neurons for retrograde cargo transport that sustains neuronal connectivity. Little, however, is known about dynein function on the postsynaptic side of the circuit. Here, we report distinct postsynaptic roles for dynein at neuromuscular junctions in *Drosophila*. Intriguingly, we show that dynein puncta accumulate postsynaptically at glutamatergic synaptic terminals. Moreover, Skittles (Sktl), a phosphatidylinositol 4-phosphate 5-kinase that produces phosphatidylinositol 4,5-bisphosphate (PIP_2_) to organize the spectrin cytoskeleton, also localizes specifically to glutamatergic synaptic terminals. Depletion of postsynaptic dynein disrupted the accumulation of Skittles and the PIP_2_ phospholipid, and organization of the spectrin cytoskeleton at the postsynaptic membrane. Coincidental with dynein depletion, we observed an increase in the size of ionotropic glutamate receptor (iGluR) fields and an increase in the amplitude and frequency of miniature excitatory junctional potentials. PIP_2_ levels did not affect iGluR clustering, nor did dynein affect the levels of iGluR subunits at the neuromuscular junction. Our observations suggest a separate, transport-independent function for dynein in iGluR cluster organization. Based on the close apposition of dynein puncta to the iGluR fields, we speculate that dynein at the postsynaptic membrane contributes to the organization of the receptor fields, hence ensuring proper synaptic transmission.

## Teaser

**Summary:** The cytoplasmic dynein motor complex has a role on the postsynaptic side of glutamatergic neuromuscular junctions that influences both the structural organization and physiology of the neuromuscular junction.

## INTRODUCTION

It is well established that microtubule motor proteins are crucial for neuronal connectivity and function. In fact, mutations in cytoplasmic dynein, the dynein co-factor dynactin, or kinesin-1 result in a number of neurodegenerative disorders (Brenner et al., 2018; Ebbing et al., 2008; Farrer et al., 2009; Inaki et al., 2010; Tsurusaki et al., 2012; Weedon et al., 2011). The onset of these neurodegenerative disorders typically follows a loss in synaptic connectivity and function between the neuron and its target tissue, often another neuron or muscle cell (Bellucci et al., 2016; Colom-Cadena et al., 2020). Investigations into the roles of dynein and kinesin motors in neuronal connectivity have largely focused on their involvement in presynaptic axonal transport. Numerous studies have elucidated the role of microtubule motors in the anterograde transport of synaptic components from the cell body to the synaptic terminal, the retrograde transport of survival signals from the synapse to the cell body, and the transport mechanisms that remove misfolded proteins and cellular debris from the axon (Bhattacharyya et al., 2002; Delcroix et al., 2003; Klopfenstein and Vale, 2004; Maday et al., 2012; Okada et al., 1995; Pack-Chung et al., 2007). Less well understood is the role of motor proteins on the postsynaptic side of neuronal connections. The majority of excitatory glutamatergic synaptic transmission in the mammalian central nervous system occurs on dendritic spines of neurons. These specialized postsynaptic compartments protrude from the dendritic branches to synapse with other neurons. Although the shafts of the dendrites are enriched in microtubules, dendritic spines are enriched in F-actin and were previously thought to be largely devoid of microtubules (Kaech et al., 1997, 2001). However, subsequent studies demonstrated that microtubules do in fact enter the dendritic spines (Gu et al., 2008; Gu and Zheng, 2009; Hu et al., 2008; Jaworski et al., 2009). Studies examining microtubule motor function in dendrites have found that both dynein and kinesin-1 act to transport AMPA glutamate receptors into dendritic shafts (Heisler et al., 2014; Kapitein et al., 2010). In turn, myosin motors are thought to transport these receptors and other cargoes along the actin cytoskeleton to the membrane of dendritic spines (Esteves da Silva et al., 2015; Wang et al., 2008). The delivery of specific subsets of cargoes to dendritic spines might involve other kinesins. For example, the transport of synaptotagmin IV requires the kinesin-3 family motor KIF1A (McVicker et al., 2016), whereas multiple related kinesin-1 proteins (e.g. KIF5A, KIF5B and KIF5C) are differentially involved in the transport of RNA-binding proteins that contribute to spine morphogenesis, density and plasticity (Zhao et al., 2020). It will be important to understand the full repertoire of microtubule-based motors in dendrites that contribute to synaptic function.

*Drosophila* type 1 neuromuscular junction (NMJ) synapses are glutamatergic, containing ionotropic glutamate receptors that are functionally equivalent to AMPA receptors of the excitatory synapses in the mammalian central nervous system. In addition to receptors, the structural, cytoskeletal and membrane-associated components that organize and regulate the postsynaptic *Drosophila* NMJ are functionally conserved with orthologous components in the postsynaptic dendritic spines of mammals. These include cell adhesion molecules that link the presynaptic and postsynaptic compartments (e.g. Neuroligins and NCAMs/Fas2), components of the postsynaptic density (e.g. PSD-95, Dlg) and regulators of the actin cytoskeleton [e.g. Adducin (also known as Hts) and α/β-Spectrins] (Banovic et al., 2010; Guan et al., 1996; Kohsaka et al., 2007; Matsuoka et al., 1998; Pielage et al., 2006; Sun et al., 2011; Wang et al., 2011). Moreover, similar to mammalian dendritic spines, the postsynaptic side of the *Drosophila* NMJ is enriched in F-actin and thought to be largely devoid of microtubules, although only a subset of microtubules has previously been examined (Coyle et al., 2004; Ramachandran et al., 2009; Ruiz-Canada et al., 2004). Given the similarities in structure and function, the *Drosophila* NMJ is an excellent model to further probe the postsynaptic function of microtubule motor proteins.

In this study, we explored the role of the microtubule motor protein, cytoplasmic dynein, at the postsynaptic side of excitatory glutamatergic synapses in *Drosophila*. We report that cytoplasmic dynein accumulates on the postsynaptic side of NMJs. Our further analysis suggests that dynein transports the phosphatidylinositol-4 phosphate 5-kinase (PI4P5K) Skittles (Sktl) to the postsynaptic NMJ, where it helps to organize the postsynaptic spectrin–actin cytoskeleton. Additionally, postsynaptic dynein is necessary for synaptic growth and the organization of glutamate receptor fields at the postsynaptic NMJ, influencing synaptic transmission.

## RESULTS

### Dynein localizes to the postsynaptic NMJ through its motor activity

**Fig. 1.**
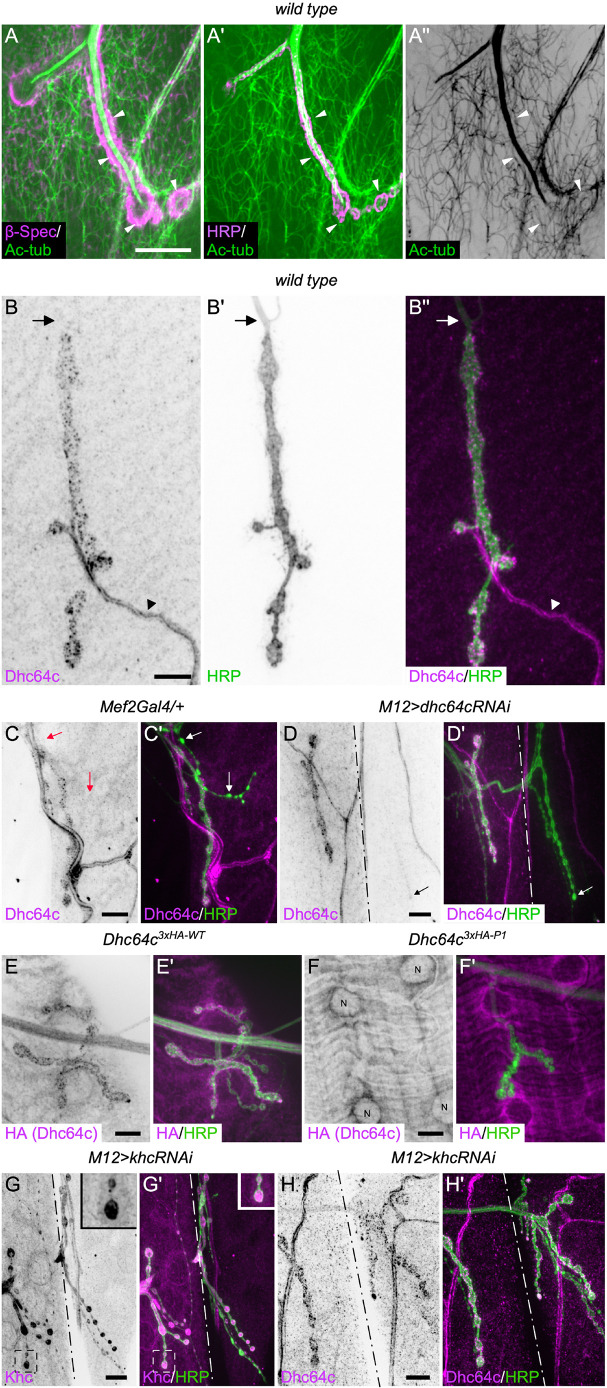
**Cytoplasmic dynein has a punctate postsynaptic localization that requires its motor function.** (A–A″) The microtubule cytoskeleton at the neuromuscular junction (NMJ) was visualized using an acetylated-α-tubulin (Ac-tub) antibody. β-Spectrin localization marks the postsynaptic side of the NMJ (A), whereas HRP labels neuronal membranes (A′). A single focal plane is shown. A subset of microtubules overlapped with postsynaptic β-Spectrin, some of which are indicated by white arrowheads. (B–Hʹ) The localizations of cytoplasmic dynein and kinesin-1 at the NMJ were assessed using antibodies that specifically recognize the dynein heavy chain subunit Dhc64c and the kinesin heavy chain subunit Khc. (B–B″) Dynein heavy chain localizes to postsynaptic puncta that are in close apposition to the neuronal membrane labeled with HRP. The arrows indicate the position of the axon that lacks dynein puncta. The arrowheads indicate a tracheal branch in the muscle that also is enriched with dynein. (C,C′) Dynein specifically localizes to glutamatergic type I synaptic terminals. The arrows indicate a type III peptidergic terminal that lacks dynein puncta. (D,D′) Depletion of dynein heavy chain in muscle 12 (right side of the dash-dotted line) demonstrates that these puncta are postsynaptic and dynein specific. The left side of the line is muscle 13 for comparison. The arrow indicates visible presynaptic dynein staining when dynein was depleted from the muscle. (E,E′) A 3×HA-tagged dynein heavy chain subunit expressed under its endogenous promoter had a similar postsynaptic punctate distribution as observed with the anti-Dhc64c antibody. (F,F′) A 3×HA-tagged dynein heavy chain subunit containing mutations in P-loop 1 (P1), required for ATP hydrolysis, did not localize to postsynaptic puncta, demonstrating the requirement of motor activity for the postsynaptic NMJ localization of dynein. Instead, dynein accumulation is visible surrounding the muscle nuclei, labelled with the letter ‘N’ (F). (G,G′) Kinesin heavy chain (Khc) was depleted from muscle 12 (right side of the dash-dotted line). Depletion of Khc in muscle 12 compared to the endogenous staining of Khc in muscle 13 (left side of the line) shows that kinesin-1 is enriched on the presynaptic side of the NMJ without an obvious postsynaptic enrichment. The boxed area for muscle 13 is shown as an inset to show kinesin-1 staining at the NMJ. (H,H′) Depletion of kinesin-1 from muscle 12 (right side of the dash-dotted line) did not affect the subcellular postsynaptic localization of dynein. Images are representative of at least six independent animals analyzed. Scale bars: 10 µm.

To investigate the postsynaptic function of motor proteins at the NMJ, we characterized the organization of the postsynaptic microtubule cytoskeleton and the distribution of the motor proteins cytoplasmic dynein and kinesin-1 within the postsynaptic muscle. Microtubules surrounded the NMJ and overlapped with a postsynaptic density protein, β-Spectrin, at the NMJ (Fig. 1A–A″; Fig. S1A–B‴), supporting a potential role for microtubule motor proteins on the postsynaptic side of NMJs. We found that cytoplasmic dynein had a unique, postsynaptic localization at NMJs that has not been previously described. The heavy chain subunit of cytoplasmic dynein (Dhc64c) localized to postsynaptic puncta in the muscle cell and in apposition to the presynaptic neuronal tissue at NMJs (Fig. 1B–B″). Intriguingly, although dynein expression in larval skeletal muscles is ubiquitous, the localized accumulation of dynein puncta is specific to glutamatergic type I synaptic terminals. We did not observe dynein accumulation at type III peptidergic terminals (Fig. 1C,C′) nor did we observe accumulation at type II octopaminergic terminals (data not shown) (Gramates and Budnik, 1999; Prokop, 2006). Other components of the dynein complex (e.g. dynein light intermediate chain or Dlic) and the associated dynactin accessory complex (e.g. p150^Glued^) are similarly localized specifically to puncta surrounding glutamatergic NMJs (Fig. S1C,C′) (Eaton et al., 2002). To confirm that the puncta we observed were specific to cytoplasmic dynein, we depleted the dynein heavy chain subunit by RNAi using the driver *M12-Gal4*, which is expressed specifically in muscle 12 (Inaki et al., 2010). We then compared the staining of dynein in muscle 12 to that in the adjacent muscle 13. In muscle 12, dynein was only present on the neuronal side of the NMJ, whereas in muscle 13, dynein puncta were observed (Fig. 1D,D′). Taken together, these results demonstrate that dynein has a unique punctate localization at glutamatergic NMJs that is muscle specific.

We speculate that the localization of dynein at the glutamatergic NMJs is due to its motor activity. To test this prediction, we examined the localization of a previously characterized Dhc64c protein containing mutations in P-loop 1 (Dhc64c^3×HA-P1^), defective in ATP hydrolysis and microtubule-based motility (Silvanovich et al., 2003). By comparison to a wild-type, epitope-tagged Dhc64c protein (Dhc64c^3×HA-WT^), the dynein mutant protein did not localize to postsynaptic puncta at the NMJ, demonstrating that dynein motor activity is required for its localization at the NMJ (Fig. 1E–F′). This result also implies that at least a subset of the microtubules within the muscle are oriented with their minus ends toward the NMJ, permitting the NMJ accumulation of dynein, a minus-end-directed motor. We hypothesize that the motor activity of dynein is important for the transport and localization of postsynaptic proteins and/or RNAs required for NMJ functionality.

The plus-end-directed motor protein kinesin-1 often functions cooperatively with cytoplasmic dynein. In particular, within neurons, dynein and kinesin-1 act in concert to regulate the transport of numerous cellular cargoes, including motor complexes themselves, up and down the axon (Lloyd et al., 2012; Martin et al., 1999; Neisch et al., 2017; Pilling et al., 2006; Twelvetrees et al., 2016). Additionally, kinesin-1 is involved in the transport of glutamatergic receptor subunits in other systems (Heisler et al., 2014; Hoerndli et al., 2013), suggesting a postsynaptic function. To test this possibility, we examined the subcellular localization of the kinesin heavy chain (Khc) subunit of kinesin-1 at NMJ using both a Khc-specific antibody and a ubiquitously expressed Khc–GFP protein. In contrast to the localization of dynein at the NMJ, we did not observe an accumulation of Khc at the postsynaptic NMJ (Fig. 1G,G′; Fig. S1D,D′). Instead, Khc was enriched within the presynaptic terminals. Moreover, depletion of Khc did not impact the postsynaptic NMJ accumulation of dynein (Fig. 1H,H′; Fig. S1E–F′), suggesting that there is no interdependence of the motor proteins for the postsynaptic localization of dynein. Our results demonstrate that dynein has a unique postsynaptic localization and function that is independent of kinesin-1 activity.

### Postsynaptic dynein is necessary for synaptic growth

During a period of rapid growth of the *Drosophila* larva, body wall muscles grow significantly, increasing in surface area by ∼100-fold. To maintain synaptic homeostasis, the presynaptic terminal must grow as well, continuously adding synaptic boutons (reviewed in Menon et al., 2013). Although the presynaptic disruption of several cytoskeletal components is known to disturb synaptic homeostasis and synaptic growth, the contribution of postsynaptic cytoskeletal elements in the regulation of synaptic growth is not well understood (Blunk et al., 2014; Chou et al., 2020; Graf et al., 2011; Mao et al., 2014; Migh et al., 2018; Pawson et al., 2008; Pielage et al., 2006; Roos et al., 2000; Sherwood et al., 2004; Zhao et al., 2013).

**Fig. 2.**
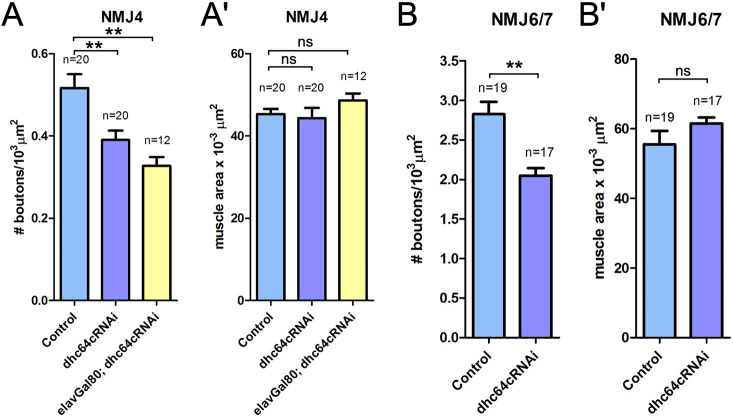
**Postsynaptic dynein modulates synaptic growth.** To probe whether postsynaptic dynein influences synaptic growth, the number of boutons and the total muscle area were measured for muscle 4 and muscles 6/7. (A) The number of boutons per muscle area at NMJ4 was significantly reduced when dynein heavy chain was depleted. However, the muscle area was not significantly affected (Aʹ). (B) Similarly, the number of boutons per muscle area at NMJ6/7 was significantly reduced when dynein heavy chain was depleted. Again, the muscle area was not significantly affected by depletion of dynein in the muscle (B′). *elav-Gal80* was expressed to verify that the changes in bouton numbers observed were not due to any nervous system expression from *Mef2-Gal4*. *n* indicates the number of NMJs analyzed. Bars show the mean±s.e.m. Unpaired two-tailed *t*-test results: ns, not significant; **P<0.005. For each genotype, ten animals were analyzed.

Given the unique pattern of postsynaptic dynein accumulation at the NMJ, we asked whether postsynaptic dynein is important for synaptic growth and NMJ morphology. We used the somatic muscle driver *Mef2-Gal4* to express an RNAi line that specifically targets the dynein heavy chain subunit (Ranganayakulu et al., 1996). To analyze the impact on NMJs, we used the presynaptic and postsynaptic membrane markers, horseradish peroxidase (HRP) and Discs large (Dlg, also known as Dlg1), respectively. We quantified the number of type 1b synaptic boutons per muscle area at NMJ4 and NMJ6/7 in third instar larvae (Han et al., 2020; Shen and Ganetzky, 2009; Xing et al., 2014). To ensure that the phenotypes observed were due to loss of postsynaptic dynein, we also co-expressed *elav-Gal80* to suppress any possible Gal4 expression within neurons when analyzing NMJ4. We found that depletion of dynein within the muscle resulted in a significant reduction in the number of boutons, while having no significant impact on muscle area (Fig. 2). Our results indicate that postsynaptic dynein impacts synaptic growth. One possible explanation for this is that dynein delivers and/or localizes one or several postsynaptic components at the NMJ to regulate synaptic terminal growth.

### Postsynaptic dynein is required for proper glutamate receptor clustering

**Fig. 3.**
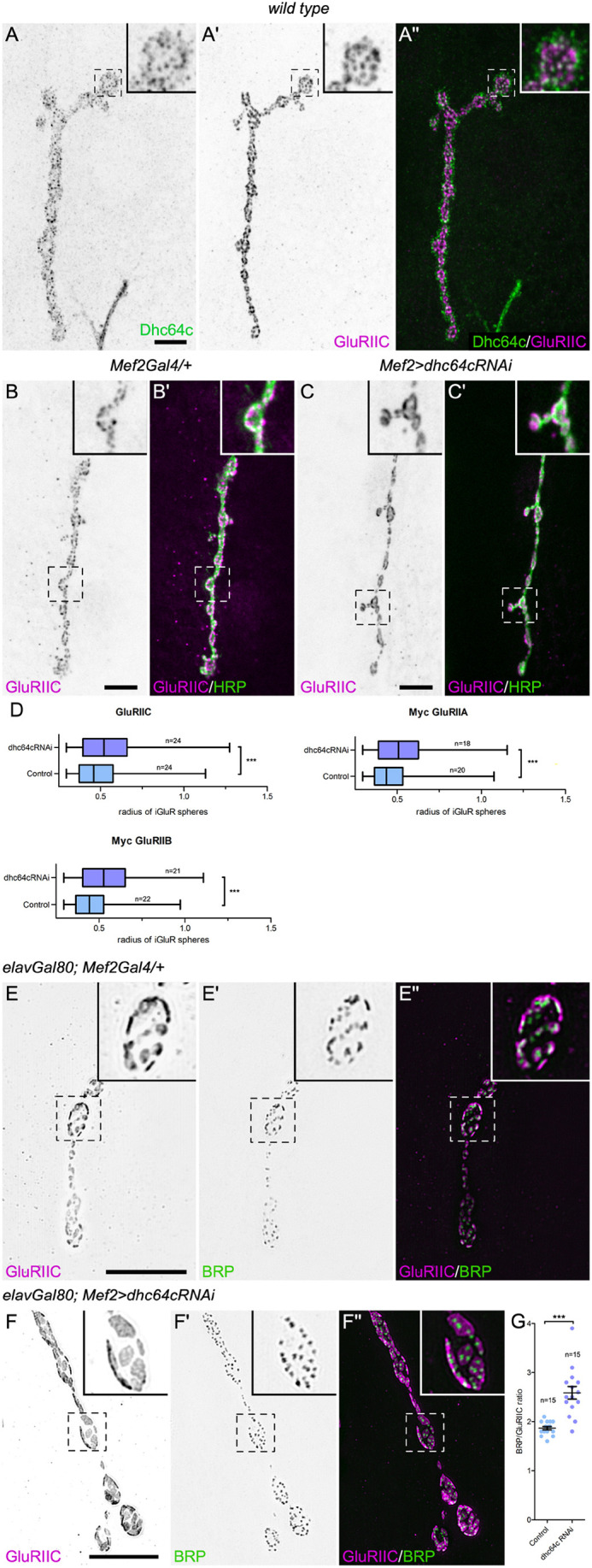
**Dynein is required for ionotropic glutamate receptor clustering.** (A–A″) The punctate distribution of dynein, although somewhat broader, was similar to that of the glutamate receptor subunit GluRIIC at the NMJ. A single optical section through the NMJ is shown. In the inset, many GluRIIC positive puncta can be seen to overlap with dynein puncta and appear white (A″). (B–D) Examples of the GluRIIC localization in a control animal (B,B′) and in a dynein-depleted animal (C,C′) are shown, which were used for the analyses in D. HRP labels the presynaptic side of the NMJ, and single optical sections through the NMJ are shown. (D) Box plots of the radii (in µm) of ionotropic glutamate receptor (iGluR) spheres in control animals, and those depleted of dynein by RNAi. Boxes show the interquartile range, whiskers show the complete range, and the median is marked with a line. *n* indicates the number of NMJs analyzed. Unpaired one-tailed *t*-test results: ***P<0.0001. (E–F″) Structured illumination microscopy images of the localization of the GluRIIC subunit and Bruchpilot (BRP), a presynaptic active zone component, in control animals (E–E″) and in animals depleted of dynein (F–F″) within the muscle. (G) Quantification of the number of BRP active zone puncta apposed to a postsynaptic glutamate receptor cluster for control animals and those depleted of muscle dynein. *n* indicates the number of NMJs analyzed and each point on the graph is an individual NMJ. Bars show the mean±s.e.m. Unpaired two-tailed *t*-test results: ***P<0.0001. Boxed areas are shown as insets. Scale bars: 10 µm. For analysis of glutamate receptor field sizes, six animals were analyzed for each genotype, except for Myc-GluRIIA in the *dhc64cRNAi* background, for which five animals were analyzed. For BRP to GluRIIC ratios, four animals were analyzed for each genotype.

The punctate localization of dynein at glutamatergic synaptic terminals resembles that of glutamate receptors at NMJs (Petersen et al., 1997; Qin et al., 2005). We compared the localization of dynein with receptor subunits and found that the distribution of dynein aligned with the pattern of glutamate receptors (Fig. 3A–A″), with some overlap (white in inset in Fig. 3A″), suggesting that dynein influences glutamate receptor localization.

*Drosophila* glutamate receptors are heteromeric tetramers composed of three essential subunits – GluRIIC, GluRIID and GluRIIE – that assemble with either the GluRIIA or GluRIIB subunit (Qin et al., 2005). We examined the effects of dynein muscle depletion on both the quantity and localization of several receptor subunits: GluRIIA, GluRIIB and GluRIIC. We found that relative to controls, the loss of dynein did not significantly impact the quantity of GluRII receptors at the NMJ (Fig. S2A). However, we observed a reorganization of the receptor fields (Fig. 3B–C′, GluRIIC; Fig. S2C–J, GluRIIA and GluRIIB). We quantified this reorganization as reflected in the receptor field size and found that there was a significant increase (Fig. 3D; Fig. S2B). These results suggest that dynein is required for the proper assembly and/or organization of the receptor fields at the NMJ.

Glutamate receptor fields on the postsynaptic side of the junction are apposed to presynaptic active zones, where the glutamate neurotransmitter is docked and released. To acquire higher-resolution images of the glutamate receptor fields and the presynaptic active zones, we used super-resolution structured illumination microscopy (SIM). We examined receptor fields in control animals and those depleted of postsynaptic dynein (Fig. 3E–F″). Glutamate receptor fields were visualized using an antibody against the GluRIIC receptor subunit and active zones were visualized using an antibody against Bruchpilot (BRP), a cytomatrix component required for active zone assembly and structural integrity (Kittel et al., 2006; Wagh et al., 2006). Other investigators have reported ∼1–1.5 BRP puncta per glutamate receptor field in control animals (Hong et al., 2020; Pielage et al., 2006; Viquez et al., 2009; Wairkar et al., 2009), suggesting ∼1–1.5 active zones per receptor cluster. In our own analysis of SIM images, we found that there were an average (±s.e.m.) of 1.87±0.04 BRP puncta per glutamate receptor cluster in control animals. Significantly, this number increased to 2.59±0.13 when dynein was depleted from the muscle (Fig. 3G). In contrast, we did not see a change in the number of BRP puncta per NMJ (Fig. S2K), suggesting that the overall number of presynaptic active zones was not changed. Taken together, these results suggest that organization of the glutamate receptor fields depends on dynein. The loss of postsynaptic dynein increases the receptor field size and impacts the relative positioning of the juxtaposed presynaptic active zones.

### Postsynaptic dynein functions in modulating spontaneous synaptic transmission

**Fig. 4.**
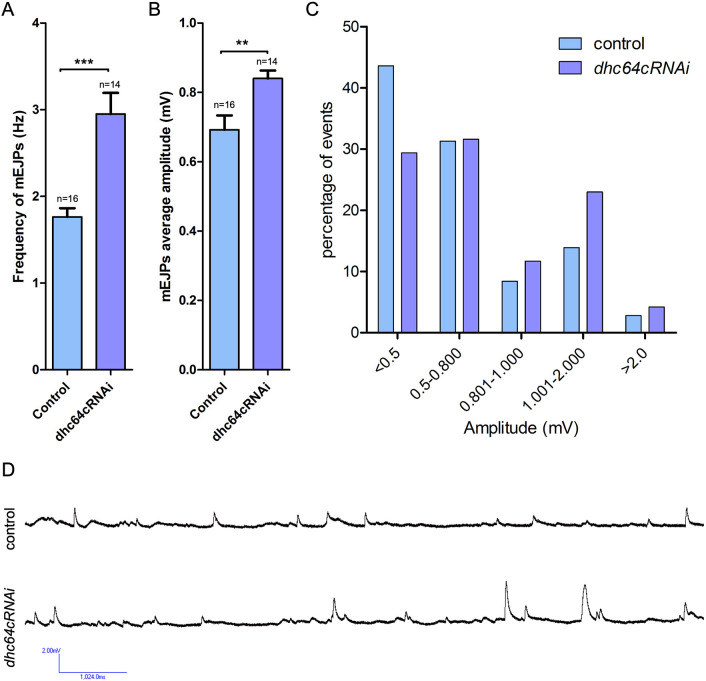
**Postsynaptic dynein is important for controlling spontaneous synaptic transmission.** Electrophysiology recordings were carried out to measure spontaneous release of glutamate containing vesicles (determined by measuring miniature excitatory junctional potentials or mEJPs) at NMJs in control animals and those depleted of dynein within the muscle. Muscle 6 of abdominal segments A2 and A3 were used for recordings. (A) The frequency of mEJPs was significantly increased when dynein was depleted from the muscle. (B) The average amplitude was also significantly increased when dynein was depleted from the muscle. (C) A graph showing the percentage distribution of events at increasing amplitudes in control and dynein-depleted animals. (D) Representative mEJP recordings from a control animal and a dynein-depleted animal. *n* indicates the number of recordings analyzed. Bars show the mean±s.e.m. Unpaired two-tailed *t*-test results: **P≤0.005; ***P<0.0001. For the control genotype, nine animals were analyzed, and for the *dhc64cRNAi* genotype, seven animals were analyzed.

To determine whether there are physiological consequences resulting from postsynaptic dynein loss, we measured the spontaneous release of glutamate (determined by measuring miniature excitatory junctional potentials or mEJPs). We found that depletion of dynein resulted in both a significant increase in the frequency of mEJPs and an increase in the amplitude of mEJPs (Fig. 4A,B,D). In control animals, there were very few spontaneous release events with large amplitudes, with only 25.1% of events having amplitudes larger than 0.8 mV. However, dynein-depleted animals exhibited a greater number of events and increased amplitudes overall, with 40% of events having an amplitude larger than 0.8 mV (Fig. 4C). Increased amplitudes of mEJPs have been observed for changes in postsynaptic glutamate receptor organization (Kittel et al., 2006; Pielage et al., 2006; Vautrin and Barker, 2003) and correlated with the changes we found in receptor clustering when dynein was depleted postsynaptically. Changes in the frequency of mEJPs is generally thought to be associated with presynaptic changes, as each peak is the result of neurotransmitter release upon fusion of a synaptic vesicle with the plasma membrane (reviewed in Bykhovskaia and Vasin, 2017). This result suggests that postsynaptic dynein contributes to transsynaptic communication at the NMJ.

### Dynein is necessary for the localization of membrane-associated postsynaptic proteins

**Fig. 5.**
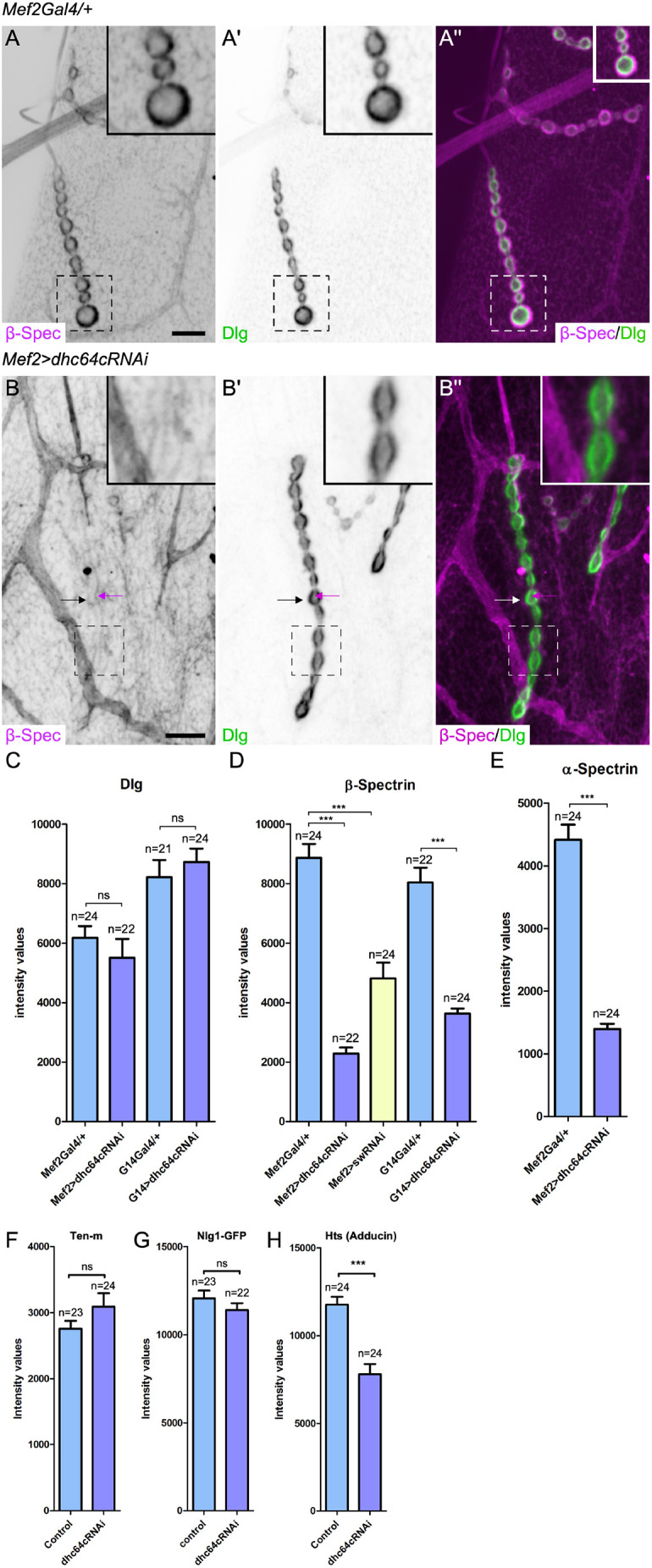
**Dynein is required to organize the postsynaptic spectrin cytoskeleton.** (A–A″) In control animals, Dlg and β-Spectrin partially overlap at the postsynaptic NMJ, with β-Spectrin localization extending beyond the localization of Dlg. (B–B″) Dynein depletion resulted in a disruption in the postsynaptic localization of β-Spectrin; however, it did not affect Dlg localization. The black and white arrows indicate the postsynaptic side of the NMJ, where a low level of β-Spectrin staining is visible. The magenta arrows indicate presynaptic β-Spectrin localization, which is not typically visible because of the greater intensity of postsynaptic β-Spectrin. (C) Quantification of the intensity of Dlg at the NMJ in controls and when Dhc64c was depleted from the muscle, using two different muscle drivers. (D) Quantification of the intensity of β-Spectrin at the NMJ in controls and when Dhc64c or the dynein intermediate chain (short wing, sw) was depleted from the muscle. (E) Quantification of the intensity of α-Spectrin at the NMJ in controls and when Dhc64c was depleted from the muscle using the *Mef2-Gal4* driver. (F–H) Quantification of Ten-m (F), Nlg1–GFP (G) and Hts (Adducin) (H) intensity levels at NMJ4. Unpaired two-tailed *t*-test results: ns, not statically significant; ***P<0.0001. *n* indicates the number of NMJs analyzed. Bars show the mean±s.e.m. In A–B″, boxed areas are shown as insets. Scale bars: 10 µm. For each genotype, six animals were analyzed.

To better understand dynein function in postsynaptic NMJ organization and physiology, we analyzed the localization of proteins that organize the postsynaptic glutamatergic NMJ. Both Dlg and β-Spectrin are specific to glutamatergic synaptic terminals, localizing to the subsynaptic reticulum, an elaboration of the postsynaptic muscle cell membrane (Featherstone et al., 2001; Guan et al., 1996; Pielage et al., 2006). In wild-type animals, postsynaptic Dlg was closely juxtaposed to presynaptic boutons, whereas β-Spectrin extended beyond the Dlg domain as visualized by immunolocalization studies (Fig. 5A–A″; Fig. S3A–B″ for anti-β-Spectrin antibody specificity). Relative to Dlg and β-Spectrin localization, we found that dynein puncta lay just inside the β-Spectrin domain (Fig. S3C–C″) and overlap with Dlg (Fig. S3D–D″).

To address whether dynein is required for the localization of postsynaptic Dlg or β-Spectrin, we depleted dynein by RNAi using the muscle drivers *Mef2-Gal4* and *G14-Gal4* (Shishido et al., 1998). Although dynein and Dlg have overlapping localizations, we found that when dynein was depleted from the muscle, there was no change in the intensity of Dlg at the postsynaptic membrane (Fig. 5B′–C). In contrast, the levels of β-Spectrin were significantly reduced when dynein was depleted (reduced by ∼55% with *G14-Gal4*, and by ∼74% with *Mef2-Gal4*; Fig. 5B,B″,D). Likewise, we found that the levels of α-Spectrin, which tetramerizes with β-Spectrin, were also significantly decreased (reduced by ∼68%; Fig. 5E). We depleted a second subunit of the dynein complex, the intermediate chain subunit short wing (sw), to confirm that loss of dynein function reduces β-Spectrin levels (reduced by 46%, Fig. 5D) and impacts the postsynaptic spectrin cytoskeleton at the NMJ.

Transmembrane cell adhesion molecules promote synapse assembly and postsynaptic organization, including the organization of the spectrin cytoskeleton. Similar to dynein, both Ten-m and Nlg1 are required for synaptic growth and organization of the spectrin cytoskeleton, but not postsynaptic Dlg localization (Banovic et al., 2010; Mosca et al., 2012; Mozer and Sandstrom, 2012; Xing et al., 2018). We asked whether dynein organizes the spectrin cytoskeleton by mediating the localization of cell adhesion molecules at the NMJ. For both Ten-m and Nlg, we observed no reduction in the levels of these proteins following depletion of postsynaptic dynein (Fig. 5F,G; Fig. S4A–K′). Our results suggest that postsynaptic dynein does not alter the trafficking and/or insertion of the transmembrane cell adhesion molecules.

We further evaluated whether dynein impacts the recruitment of the spectrin-associated protein Adducin. Adducin is a cytoskeletal protein that links α/β-Spectrin tetramers to actin filaments (Gardner and Bennett, 1987; Mische et al., 1987). We found that the levels of postsynaptic Adducin were significantly reduced when dynein was depleted from the muscle (by ∼34%; Fig. 5H; Fig. S3E–F′). Thus, dynein might impact the localization of the spectrin cytoskeleton by modifying the levels of Adducin at the NMJ, either through trafficking of Adducin or regulating an upstream factor that is required for Adducin localization at the NMJ.

### Dynein is required for the localization of Skittles and PIP_2_ production at the NMJ

The phospholipid membrane component phosphatidylinositol 4,5-bisphosphate (PIP_2_) is known to accumulate postsynaptically at the NMJ (Wang et al., 2014). PIP_2_ binds spectrin and helps localize it to the membrane by enhancing spectrin–actin interactions (Das et al., 2008). In addition, PIP_2_ regulates Adducin localization at the postsynaptic membrane, similarly enhancing the binding of Adducin to actin and impacting signaling.

**Fig. 6.**
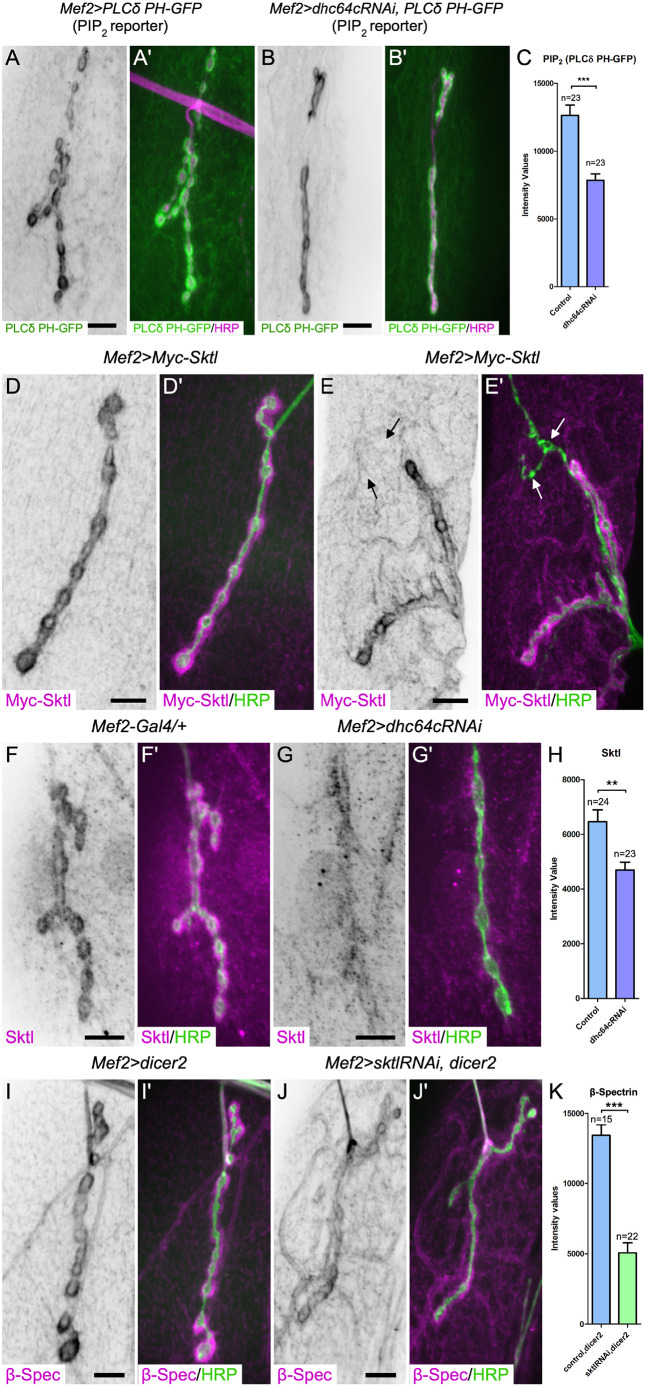
**Dynein is essential for the localization of Skittles kinase to the NMJ for production of PIP_2_ locally at the postsynaptic NMJ membrane.** (A–C) To examine the distribution of phosphatidylinositol 4,5-bisphosphate (PIP_2_) at the postsynaptic NMJ membrane, the PIP_2_ reporter *PLCδ-PH-GFP* was expressed in the muscle. (A,A′) PIP_2_ localization is enriched at the postsynaptic membrane. (B,B′) Depletion of dynein in the muscle reduced postsynaptic PIP_2_ membrane enrichment. (C) Quantification of the postsynaptic membrane intensity of PIP_2_ (PLCδ-PH-GFP) in control animals and those depleted of dynein by RNAi. *n* indicates the number of NMJs analyzed. Six animals were analyzed for each genotype. (D,D′) 6×Myc-tagged Skittles (Sktl) expressed in the muscle is enriched at the postsynaptic NMJ. (E,E′) Skittles localization is specific to glutamatergic type I synaptic terminals. The arrows indicate a type III peptidergic terminal that lacks Skittles. (F,Fʹ) A Skittles-specific antibody shows a similar postsynaptic localization pattern at the NMJ. (G,Gʹ) The postsynaptic localization of Skittles was lost when dynein was depleted from the muscle. (H) Quantification of the postsynaptic membrane intensity of Skittles in control animals and those depleted of dynein by RNAi. Six animals were analyzed for each genotype. (I–Jʹ) Postsynaptic β-Spectrin levels in *dicer2*-expressing control animals (I,Iʹ) and those depleted of Skittles (J,Jʹ). (K) Quantification of the postsynaptic membrane intensity of β-Spectrin in control animals and those depleted of Skittles by RNAi shows a statistically significant decrease in Skittles-depleted animals. Six animals were analyzed for *sktlRNAi,dicer2* and four animals for *dicer2*. *n* indicates the number of NMJs analyzed. Bars show the mean±s.e.m. Unpaired two-tailed *t*-test results: **P<0.005; ***P<0.0001. In all images, HRP labels the presynaptic side of the NMJ. Scale bars: 10 µm.

To assess whether dynein impacted PIP_2_, we examined the levels of PIP_2_ at the postsynaptic membrane using a reporter that binds PIP_2_ (Li et al., 2020; Tan et al., 2014; Wang et al., 2014). Depletion of dynein in the muscle significantly reduced the levels of PIP_2_ at the postsynaptic membrane (by ∼38%, Fig. 6A–C). This reduction in PIP_2_ correlated with postsynaptic reduction in Adducin and β-Spectrin levels (Fig. 5) and might follow from reduced binding of Adducin and β-Spectrin to actin (Das et al., 2008; Gardner and Bennett, 1987; Mische et al., 1987; Wang et al., 2014).

PIP_2_ is produced by the conversion of phosphatidylinositol 4-phosphate (PI4P) into PIP_2_ by PI4P5Ks. In *Drosophila*, one of the PI4P5Ks is Skittles (Chakrabarti et al., 2015; Hassan et al., 1998). Skittles can form a complex with the light intermediate chain subunit of dynein (Jouette et al., 2019). Intriguingly, we report for the first time that, similar to the postsynaptic localization of dynein, Myc-tagged Skittles also localizes specifically to glutamatergic type I synaptic terminals (Fig. 6D–E′). Moreover, using an antibody specific to Skittles, we confirmed that the PI4P5K is localized on the postsynaptic side of type I terminals (Fig. 6F,F′). This localization was disrupted by the loss of dynein (reduced by ∼27%; Fig. 6G,H). As expected, reducing the levels of postsynaptic Skittles reduced the PIP_2_ reporter levels at the postsynaptic NMJ (Fig. S5A). We speculate that postsynaptic PIP_2_ binds Adducin and/or β-Spectrin to mediate the organization of the spectrin cytoskeleton. To test this model, we asked whether Skittles was required at the NMJ for β-Spectrin postsynaptic organization. We observed a significant decrease in the level of postsynaptic β-Spectrin (reduced by ∼62%; Fig. 6I–K) when Skittles was depleted in the muscle using an effective RNAi (Fig. S5B–C″). Our observations are consistent with a model in which dynein actively transports Skittles to the postsynaptic NMJ, where it locally facilitates PIP_2_ production. In turn, PIP_2_ contributes to organizing the postsynaptic spectrin cytoskeleton.

### Dynein mediates glutamate receptor organization independent of Skittles

**Fig. 7.**
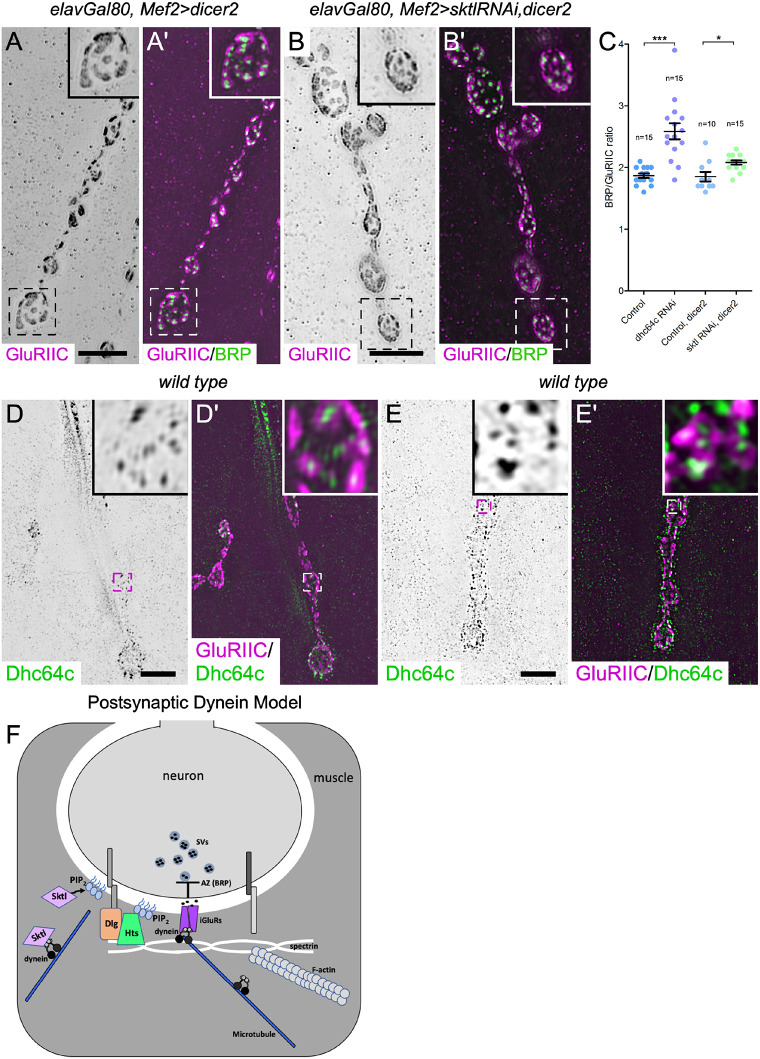
**Skittles does not substantially contribute to glutamate receptor clustering, whereas dynein puncta localize to the center of glutamate receptor fields.** (A–Bʹ) Structured illumination microscopy was used to image and analyze GluRIIC fields and their apposition to presynaptic BRP puncta. GluRIIC receptor cluster size, and their apposition to presynaptic Bruchpilot (BRP) puncta appeared to be similar in controls (A,A′) and Skittles-depleted animals (B,B′). Boxed areas are shown as insets. (C) Quantification of the number of BRP active zone puncta apposed to a postsynaptic glutamate receptor cluster for control animals and those depleted of postsynaptic Skittles was compared to the data shown in Fig. 3G for dynein depletion, replicated again here for a side-by-side comparison. *n* indicates the number of NMJs analyzed and each point on the graph is an individual NMJ. Bars show the mean±s.e.m. Unpaired, two-tailed *t*-test results: *P<0.05; ***P<0.0001. For BRP to GluRIIC ratios, three animals were analyzed for the *dicer2* control and four animals were analyzed for *sktlRNAi,dicer2*. (D–E′) Structured illumination microscopy was used to image glutamate receptor fields and dynein together at NMJs to better determine the relationship between the two components. Two examples of wild-type synaptic terminals are shown. GluRIIC labels glutamate receptor fields and Dhc64c labels the dynein heavy chain. In the merged image insets, dynein often localizes to the center of the glutamate fields (D′,E′). Images in D and E are representative of five independent animals analyzed. Scale bars: 10 µm. (F) A model for how dynein functions on the postsynaptic side of the NMJ to transport Skittles (Sktl) for local production of PIP_2_, organization of the postsynaptic spectrin cytoskeleton, and stabilization and/or organization of glutamate receptor fields. AZ, active zone; iGluRs, ionotropic glutamate receptors; SVs, synaptic vesicles; Sktl, Skittles; PIP2, phosphatidylinositol 4,5-bisphosphate; BRP, Bruchpilot; Dlg, Discs large; Hts, Hu li tai shao, also known as Adducin.

Both PIP_2_ and phosphatidylinositol-(3,4,5)-triphosphate (PIP_3_) phospholipids have been implicated in the localization of AMPA receptors at the postsynaptic membrane (Arendt et al., 2010; Seebohm et al., 2014). As dynein is required for both the localization of Skittles at the NMJ and proper glutamate receptor organization, we tested whether membrane levels of Skittles, and hence PIP_2_, are required to organize glutamate receptor fields. We depleted Skittles from the muscle and examined the receptor field size. In contrast to dynein depletion, the depletion of Skittles did not result in obviously enlarged glutamate receptor fields (compare Fig. 3E,F to Fig. 7A,B). Furthermore, when examining the ratio of presynaptic active zones (BRP puncta) to glutamate receptor fields, we found that in control animals expressing only *dicer2*, there were on average 1.85±0.07 BRP puncta per glutamate receptor field, similar to what we observed in our previous control samples (1.87±0.04 BRP puncta, Fig. 7C). When we depleted Skittles, we observed only a slight increase to 2.08±0.03 BRP puncta per receptor cluster. We observed a greater significant increase in receptor field size when dynein was depleted (2.59±0.13 BRP puncta, Fig. 3G). Considering that Skittles membrane levels are reduced by ∼27% upon the depletion of dynein, we infer that the enlarged glutamate receptor fields observed when dynein was depleted were not primarily a result of reduced postsynaptic Skittles, as targeted depletion of Skittles did not result in the same or a stronger phenotype.

Consistent with a lack of a significant alteration in the GluRII receptor field size, and in contrast to postsynaptic loss of dynein (Fig. 4), the loss of Skittles does not perturb synaptic transmission (Hassan et al., 1998). Taken together, we speculate that dynein that accumulates at the postsynaptic membrane is involved in regulating the assembly of the receptor field. Depletion of dynein at the membrane results in the aberrant increase in the receptor field size and synaptic transmission. Independently, dynein also acts in the localization of Skittles to the NMJ and, through PIP_2_, helps to organize the postsynaptic spectrin cytoskeleton.

Although our results suggest that dynein affects Skittles and in turn the spectrin cytoskeleton, it is possible that dynein impacts both Skittles and PIP_2_, and β-Spectrin separately, to organize glutamate receptor fields. Previous work reported that depletion of postsynaptic β-Spectrin results in larger glutamate receptor fields (Pielage et al., 2006). One caveat to the experiments in Pielage et al. (2006) is that β-Spectrin was depleted from the whole muscle cell and not solely from the postsynaptic membrane. β-Spectrin is known to form an intricate network throughout the muscle. We observed disorganization of the microtubule cytoskeleton throughout the muscle when β-Spectrin was depleted, suggesting that the spectrin cytoskeleton in the muscle cell assists in organizing the microtubule cytoskeleton (Fig. S6B–E⁗). We also observed a significantly decreased accumulation of dynein at the postsynaptic membrane (Fig. S6E). Taken together, these results suggest that muscle-specific β-Spectrin contributes to the organization of glutamate receptors through the microtubule network and dynein.

To better understand how dynein might contribute to the organization of glutamate receptors at the NMJ, we analyzed the localization of the dynein heavy chain subunit Dhc64c and the glutamate receptor subunit GluRIIC using SIM. As we observed previously, the GluRIIC staining often forms donut-like structures. Remarkably, dynein localized to the membrane, often at the center of the glutamate receptor fields (Fig. 7D–Eʹ). The localization was very similar to that of BRP, which localizes to the active zone, but on the presynaptic side of the junction at the center of the glutamate receptor fields (Figs 3E″ and 7A′). This pattern of localization, together with the increased size of receptor fields that results following postsynaptic depletion of dynein, suggests that dynein acts to position the localization of glutamate receptors on the postsynaptic side of the NMJ.

## DISCUSSION

Neuronal connectivity, maintained through presynaptic and postsynaptic connections, is crucial for cognitive and motor functions. Mutations within the motor protein complex, cytoplasmic dynein and its accessory complex dynactin are linked to a number of neurological disorders (Becker et al., 2020; Chevalier-Larsen et al., 2008; Fiorillo et al., 2014; Hoang et al., 2017; Honda et al., 2018; Konno et al., 2017; Lloyd et al., 2012; Moughamian and Holzbaur, 2012; Shen et al., 2018). Although the role of microtubule motor proteins in neuronal transport and synaptic function is widely recognized, the postsynaptic roles of these proteins have been largely understudied. Here, we uncover postsynaptic functions for cytoplasmic dynein in synaptic growth, the organization of glutamate receptor fields and the organization of the postsynaptic spectrin cytoskeleton at the NMJ. Together, our results suggest a model in which dynein has multiple functions at the postsynaptic NMJ. First, dynein acts to transport Skittles to the membrane, driving local PIP_2_ production, which in part contributes to the organization of the underlying spectrin cytoskeleton. Second, dynein organizes glutamate receptor fields at the membrane, potentially acting as a tether to stabilize their localization (Fig. 7F) and resulting in an impact on transsynaptic communication.

### Postsynaptic dynein versus kinesin-1 at the NMJ

We discovered that postsynaptic dynein localization is specific to synaptic terminals that release the excitatory neurotransmitter glutamate. Conversely, we did not observe enrichment of the conventional kinesin, kinesin-1, at the postsynaptic NMJ. Kinesin-1 depletion in the muscle also did not affect the localization of dynein at the postsynaptic NMJ. In addition to kinesin-1, there are 25 kinesin or kinesin-like proteins in *Drosophila* (Goshima and Vale, 2003; http://cb.m.u-tokyo.ac.jp/KIF/KIFlist.html). Whether one of these other kinesins functions cooperatively with dynein in transport at the postsynaptic NMJ remains to be determined.

### Dynein as a regulator of synaptic terminal growth

The rapid skeletal muscle growth that occurs during larval development is accompanied by coincident synaptic terminal growth to maintain stable physiological function. We found that dynein is required for growth of the synaptic terminal (Fig. 2). Several components, both presynaptic and postsynaptic, display transsynaptic influences and can affect synaptic NMJ growth. These include cytoskeletal components, cell adhesion molecules and signaling components (Banovic et al., 2010; Koch et al., 2008; Li et al., 2007; Marques et al., 2002; McCabe et al., 2003; Miech et al., 2008; Mozer and Sandstrom, 2012; Packard et al., 2002; Pielage et al., 2006). Although morphological plasticity, such as NMJ terminal bouton number, and physiological plasticity, such as the regulation of neurotransmitter release, are thought to be linked, recent studies have revealed that this is not always the case (Borczyk et al., 2019; Goel et al., 2019; Kidd and Lieber, 2016). When dynein was depleted postsynaptically, we saw both synaptic growth defects and physiological defects in spontaneous synaptic transmission. This contrasts with a previous study that showed that muscle disruption of the dynactin complex, using expression of a p150^Glued^ dominant-negative construct, did not reduce bouton numbers (Eaton et al., 2002). There are two possible explanations for these observed differences. First, the Eaton et al. (2002) study used the muscle driver *Mhc-Gal4* that expresses late in development. Moreover, a dominant-negative form of dynactin (*p150^Glued^*) was expressed to inhibit dynein functions. The late expression of the dominant negative dynactin protein and/or the mechanism of dominant negative dynactin might not have inhibited all functions of the endogenous dynein–dynactin complex to the same extent as the RNAi strategy that we used.

The mechanism for the impact of dynein on synaptic terminal growth is yet to be determined. One possible mechanism is through the impact of dynein on PIP_2_ levels at the membrane. We have shown that depletion of dynein results in the reduction of PIP_2_ at the postsynaptic membrane (Fig. 6). Among the many functions of PIP_2_, its role in exocytosis of secretory vesicles is crucial in numerous physiological functions. For example, on the presynaptic side of neuronal connections, the exocytosis of neurotransmitter-containing vesicles is dependent on PIP_2_ (Bradberry et al., 2019; Grishanin et al., 2004). Additional work in MCF-7 cells has shown that PIP_2_ and some of the isoforms of PI4P5K transiently accumulate specifically at sites of exocytosis (Stephens et al., 2020). In turn, accumulation of PIP_2_ and PI4P5K (i.e. Skittles) might occur during postsynaptic exocytosis of Gbb, a BMP ligand. The BMP pathway is the major retrograde signaling pathway implicated in synaptic terminal growth at the NMJ (Hoover et al., 2019; McCabe et al., 2003). Loss of muscle-derived Gbb results in undergrowth of the synaptic terminal (McCabe et al., 2003). The reduction of PIP_2_ levels following dynein depletion might affect exocytosis of Gbb or another unidentified molecule from the muscle that is important for synaptic growth.

The impact of PIP_2_ on postsynaptic exocytosis might reflect its role as an important regulator of actin cytoskeleton dynamics. PIP_2_ interacts with multiple actin-binding domain-containing proteins and promotes F-actin assembly, potentially driving the extension of the plasma membrane and exocytosis (reviewed in Katan and Cockcroft, 2020; Miklavc and Frick, 2020). Moreover, defects in the spectrin and actin cytoskeletons are common features of mutants exhibiting reduced synaptic terminal growth. For example, postsynaptic loss of each of the cell adhesion molecules Nlg1 and Ten-m results in both a synaptic terminal growth defect and a reduction in α/β-Spectrin (Mosca et al., 2012; Xing et al., 2018). In this study, we observed a correlation between reduced synaptic growth and a reduction in the levels of the F-actin-binding proteins β-Spectrin, α-Spectrin and Adducin following the depletion of postsynaptic dynein (Fig. 5). Consistent with these results, loss of postsynaptic β-Spectrin also results in decreased bouton numbers at the synaptic terminal (Pielage et al., 2006). Our results show that dynein is necessary for PIP_2_ accumulation at glutamatergic NMJs, and we hypothesize that the differential affinities of distinct actin-binding proteins for PIP_2_ (Senju et al., 2017) in turn might regulate the underlying actin cytoskeleton and exocytosis. It will be important in the future to determine whether homeostatic adaptation mechanisms can compensate for the synaptic terminal growth defects observed for postsynaptic dynein depletion.

### Skittles trafficking modulates PIP_2_ functions at the NMJ

Localization of the PI4P5K Skittles and its specific role at the excitatory glutamatergic NMJs has not previously been described. Our results show that Skittles localizes specifically to the postsynaptic side of glutamatergic synaptic terminals, and that this localization is dependent on dynein (Fig. 6). Taken together, the localization of Skittles and accumulation of PIP_2_ (Wang et al., 2014), suggests that Skittles functions locally at the postsynaptic membrane to produce PIP_2_ at glutamatergic NMJs. PIP_2_, which primarily localizes to the plasma membrane, can be produced from either the precursor PI4P by PI4P5Ks or the precursor phosphatidylinositol 5-phosphate (PI5P) by phosphatidylinositol 5-phosphate 4-kinases (PI5P4Ks). Interestingly, these kinases appear to produce distinct pools of PIP_2_ that carry out different cellular functions (Kolay et al., 2016; Mayinger, 2012). There is significant evidence for the actions of PIP_2_ at presynaptic sites, but knowledge of PIP_2_ function postsynaptically is limited. However, recent work suggests that PIP_2_ is a substrate for generation of inositol triphosphate (IP_3_) and diacylglycerol (DAG), key signaling elements underlying homeostatic plasticity in neurons (James et al., 2019). Thus dynein-mediated transport and the localization of Skittles to the postsynaptic membrane of glutamatergic synapses might impact IP_3_/DAG signaling and affect transsynaptic signaling.

PIP_2_ also interacts with multiple membrane and cytosolic proteins to regulate several cellular processes in neurons. As discussed above, actin regulatory proteins might be differentially recruited to localized pools of PIP_2_ to control cytoskeletal polymerization and organization, which in turn can facilitate postsynaptic exocytosis involved in retrograde signaling. Furthermore, similar to the well-characterized role for PIP_2_ in the recycling of synaptic vesicles at presynaptic sites, the accumulation of PIP_2_ might also facilitate endocytosis at postsynaptic sites. Regulated trafficking and control of receptor abundance at the synaptic membrane is proposed to contribute to activity-induced changes in synaptic transmission (Bredt and Nicoll, 2003; Malinow and Malenka, 2002; Newpher and Ehlers, 2008). In this regard, dynein transport of Skittles might modulate local PIP_2_ levels and thus regulate the endocytosis of receptor subunits.

### Cortical dynein in the organization of GluRII fields

We observed that loss of dynein resulted in enlarged glutamate receptor fields. Previous reports have shown enlarged GluRII receptors fields when β-Spectrin is depleted (Pielage et al., 2006). Although we observed a reduction in β-Spectrin at the postsynaptic membrane when dynein was depleted, our data suggest that loss of β-Spectrin in the dynein-depleted background was not the major contributor to the enlarged glutamate receptors. First, we observed comparable knockdown levels of β-Spectrin when either dynein or Skittles was depleted; however, dynein loss had a much stronger impact on the enlargement of receptor fields compared to Skittles depletion (Fig. 7C). Second, the β-Spectrin network is found throughout the muscle, not just at the postsynaptic membrane. Pielage et al. (2006) observed larger glutamate receptor fields when β-Spectrin was depleted throughout the muscle. We found that depletion of β-Spectrin throughout the membrane resulted in disruption of the microtubule cytoskeleton and a reduction in the localization of dynein at the postsynaptic membrane (Fig. S6). These changes in the cytoskeleton in turn impact postsynaptic dynein and might contribute to the disorganization of glutamate receptor fields. Third, others have reported disruption of the spectrin cytoskeleton at the postsynaptic membrane in different genetic backgrounds without observing changes in glutamate receptor field sizes (Christophers et al., 2024; Restrepo et al., 2022; Wang et al., 2018). Taken together with these observations, our results suggest that dynein has a novel function in organizing glutamate receptor fields that is independent of Skittles/PIP_2_ and β-Spectrin.

At the NMJ, dynein localizes as puncta at the cortex of the postsynaptic cell membrane. These dynein puncta are closely associated with the center of glutamate receptor fields that are arranged in a defined pattern, suggesting that the dynein motor complexes act as a novel tether to organize glutamate receptors (Fig. 7F). Our results demonstrate that cortical dynein functions in the organization of the GluRII receptor fields at the postsynaptic membrane. Numerous cellular and developmental studies have implicated cortical dynein in the tethering of microtubule plus ends and spindle orientation that underlies establishment of cell polarity, as well as the remodeling of cellular junctions during morphogenesis (di Pietro et al., 2016; Kotak et al., 2012; Ligon and Holzbaur, 2007; Lu and Prehoda, 2013; Rodriguez-Garcia et al., 2018). For example, in the case of epithelial adherens junctions, dynein localized at sites of cell–cell contact pulls on microtubules and the cytoskeleton to organize the interface between cells and to polarize the transport of cellular factors required at the specialized membrane junctions (Ligon and Holzbaur, 2007; Ligon et al., 2001). Similar to the adherens junctions, the immunological synapse (IS) is another site of cell–cell contact where dynein functions as a cortical tether (Combs et al., 2006; Hashimoto-Tane et al., 2011; Martin-Cofreces et al., 2008; Martin-Cofreces and Sanchez-Madrid, 2018). The IS exhibits several parallels to the *Drosophila* NMJ. At both the IS and the NMJ, dynein functions to organize the proper clustering of receptors in the membrane, and this organization is critical for proper receptor signaling (this work; Hashimoto-Tane et al., 2011). At the IS, PIP_2_ is cleaved to generate IP_3_ and DAG. Strikingly, DAG subsequently binds to and recruits dynein to the T cell membrane of the IS (Liu et al., 2013; Quann et al., 2009). Intriguingly, at the NMJ, PIP_2_ is also cleaved and has been reported to produce the canonical second messengers DAG and IP_3_ that are integral to presynaptic homeostatic potentiation (James et al., 2019). We suggest that similar to the IS, DAG produced from PIP_2_ at the postsynaptic NMJ recruits dynein to the membrane.

Although we report for the first time a postsynaptic role for dynein in *Drosophila* NMJs, dynein has previously been known to bind to mammalian NCAM180 (an isoform of NCAM1), a homophilic neural cell adhesion molecule localized on both sides of neurological synapses. Dynein interacts with NCAM180 and this interaction increases cell–cell adhesion and tethering of plus-end microtubules (Perlson et al., 2013). Moreover, when the NCAM180–dynein interaction is disrupted, the density of active synapses is decreased, but the individual synapses are also much broader (Perlson et al., 2013). This broadening of the synapses might be similar to what we observed as an enlargement of the glutamate receptor fields, but on the postsynaptic side at the *Drosophila* NMJ. Thus, NCAM180 is an alternative partner for recruiting dynein to the synapse. The *Drosophila* Fas2 isoforms are orthologous to the NCAMs present at the mammalian neurological synapse (Beck et al., 2012; Neuert et al., 2020) and might similarly direct the cortical recruitment of dynein that in turn interacts with and organizes receptor fields of the postsynaptic membrane.

Our work has revealed novel postsynaptic dynein functions in synaptic terminal growth, glutamate receptor organization and the regulation of the postsynaptic cytoskeleton. To elucidate how dynein acts in these novel functions will require live-imaging studies that characterize the dynamics of the processes and how they are disrupted by the absence of postsynaptic dynein. It will be important to determine when dynein is initially localized to the NMJ and whether the postsynaptic accumulation of dynein precedes the establishment of newly formed glutamate receptor fields. Our studies further suggest novel roles for dynein in regulating PIP_2_ production and signaling. The deconstruction of the signaling cascades and physical interactions that regulate the postsynaptic cytoskeleton, synaptic terminal growth and receptor organization are important challenges in understanding the mechanisms involved in synaptic function, plasticity and related diseases of the nervous system.

## MATERIALS AND METHODS

### *Drosophila* lines

Flies were raised on standard food and all crosses were performed at 29°C. *w^1118^* was used as the wild-type outcross control. Stocks used from the Bloomington *Drosophila* Stock Center are as follows: *UAS-dhc64cRNAi^HMS01587^*, *UAS-swRNAi^HM05249^*, *UAS-BSpecRNAi^GL01174^*, *Mef2-Gal4.R*-3, *UAS-sktlRNAi^JF02796^*, *UAS-PLCdelta-PH-EGFP-3*, *mhc-GluRIIA-Myc-*2 and *mhc-GluRIIB-Myc*-3. Stocks used from Vienna *Drosophila* Resource Center (VDRC) are as follows: *UAS-khcRNAi^GD12278^* (44337). Other stocks used in this study are: *G14-Gal4/CyoGFP* (Saitoe et al., 2001), *Dhc64c::Dhc64c^3xHA-WT^* and *Dhc64c::Dhc64c^3xHA-P1^* (Silvanovich et al., 2003), *UASp-6xMyc-sktl* (Jouette et al., 2019; provided by Sandra Claret, Intitut Jacques Monod, Paris, France), *elav-Gal80* (Yang et al., 2009), *UAS-Nlg1-GFP* (Banovic et al., 2010; provided by Hermann Aberle, Heinrich Heine University of Dusseldorf, Dusseldorf, Germany), *C57-Gal4*, *UAS-DlgS97-GFP* (Bachmann et al., 2004), and *M12-Gal4*, also known as *5053A-Gal4* (Inaki et al., 2010). The genotype analyzed for each figure is provided in Table S1. Also noted in this table, if the figure is an image, is the image type shown (single section, sum of sections or maximum-intensity projection of sections).

### Immunostaining

Wandering third instar larvae were dissected in HL3 saline (70 mM NaCl, 5 mM KCl, 20 mM MgCl_2_, 10 mM NaHCO_3_, 5 mM trehalose, 11 mM sucrose and 5 mM HEPES, pH 7.2) by filleting the larvae open in Sylgard-coated dishes. Animals were fixed in 4% paraformaldehyde for 20 min for all antibodies except for anti-GluRIIA. For anti-GluRIIA antibody staining, larvae were fixed in Bouin's solution (Sigma-Aldrich) for 5 min. The same antibody solutions were used on genotypes for comparisons. Antibodies were used at the following concentrations: rat anti-HA at 1:400 (3F10, Sigma-Aldrich), mouse anti-Myc at 1:2000–1:3000 (9B11, Cell Signaling Technology), Alexa Fluor 488-conjugated mouse anti-acetylated α-tubulin at 1:50 (6-11B-1, Santa Cruz Biotechnology), mouse anti-Dhc64c at 1:400 (P1H4, McGrail and Hays, 1997), mouse anti-Dlg at 1:500 [4F3-c, Developmental Studies Hybridoma Bank (DSHB), Iowa City, IA, USA], mouse anti-GluRIIA at 1:10 (8B4D2-s, DSHB), rabbit anti-GluRIIC at 1:3000 (Ramos et al., 2015; provided by Mihaela Serpe, NIH, Bethesda, USA), rabbit anti-GluRIIB at 1:1000 (Ramos et al., 2015; provided by Mihaela Serpe), rabbit anti-Skittles 1:800 (Claret et al., 2014; provided by Sandra Claret), mouse anti-α-Spectrin at 1:10 (3A9-s, DSHB), guinea pig anti-β-Spectrin at 1:1000 (this study, see ‘β-Spectrin antibody production’ section below), rabbit anti-GFP at 1:1000 (TP401, Torrey Pines Biolabs, Secaucus, NJ, USA), mouse anti-BRP nc82-s at 1:25 (DSHB), mouse anti-Adducin at 1:100 (1B1-c, DSHB), mouse anti-Ten-m at 1:10 (mAb20-s, DSHB), rabbit anti-Kinesin at 1:200 (Kin01, Cytoskeleton, Inc., Denver, CO, USA), rabbit anti-Nlg1 at 1:500 (Banovic et al., 2010; provided by Stephan Sigrist, Freie Universität Berlin, Berlin, Germany), guinea pig anti-Nlg1 at 1:250 (Mozer and Sandstrom, 2012; provided by Brian Mozer, NIH, Bethesda, USA), mouse anti α-tubulin at 1:100 (DM1A, Sigma-Aldrich) and rat anti-tyrosinated α-tubulin at 1:100 (YL1/2, EMD Millipore). Alexa Fluor 488-, 594-, 647- or 405-conjugated goat anti-HRP antibodies at 1:1000 were used to label the presynaptic side of the junction (Jackson ImmunoResearch Laboratories; Jan and Jan, 1982). Secondary antibodies conjugated with Alexa Fluor 488 or Alexa Fluor 594 were used at 1:500 (Jackson ImmunoResearch Laboratories). Samples were mounted in Prolong Glass antifade mounting medium (Thermo Fisher Scientific).

### Bouton number per muscle area quantification

Wandering third instar larvae from crosses of *Mef2-Gal4* to *w^1118^* or *UAS-dhc64cRNAi^HMS01587^* were dissected and processed as described in the ‘Immunostaining’ section above. Images were acquired on a Zeiss Cell Observer spinning-disk confocal microscope (Zeiss, Obekochen, Germany) using a Photometrics QuantEM 512SC camera and laser lines at 488 and 561 nm, and the emitted fluorescence was collected through 503–538 nm and 580–653 nm emission filters. Zen2 software (Zeiss) was used to acquire the images. A 10× EC Plan NeoFluor 0.3NA objective (1.33 µm pixel size) was used to image muscles for area quantifications. A 63× Plan Apo 1.4NA objective (0.21 µm pixel size) was used to image the NMJ at abdominal segment A2 of muscles 6/7 and abdominal segment A3 of muscle 4. When necessary, tiling of the NMJs was done and images were stitched together using the Zen2 software. Ten female animals of each genotype were dissected and imaged. The total muscle areas were obtained by tracing the outline of the muscle in Fiji. Boutons were identified by eye and counted for NMJ6/7 and 1b boutons were counted for NMJ4.

### Quantification of protein intensities at NMJs

Wandering third instar larvae from crosses were dissected and processed as described in the ‘Immunostaining’ section above. Six female animals for each genotype were used. Animals were stained with the same primary and secondary antibody solutions and imaged under the same conditions. Samples were imaged in a Zeiss Cell Observer spinning-disk confocal microscope with a Yokogawa CSU-X1 spinning-disk confocal head (Yokogawa, Tokyo, Japan). Illumination was provided with lasers of 488 and 561 nm and the emitted fluorescence was collected through 503–538 nm and 580–653 nm emission filters, respectively. Zen2 software, a QuantEM 512SC camera (Photometrics, Tuscon, AZ, USA) and a 63× Plan Apo 1.4NA objective (0.21 µm pixel size) with a *z*-step size of 0.24 µm were used to acquire the images. NMJ images were acquired at abdominal segments A3 and A4 of muscle 4. For analysis, the last bouton of a synaptic terminal was selected. A Jython script was written for FIJI to measure the postsynaptic protein intensities at NMJs. Briefly, a 47×47 pixel area (9.96 μm×9.96 μm) was selected from the maximum-intensity projection. The selected area was used to crop all slices in the original image, creating a three-dimensional (3D) stack. Each channel underwent background correction by subtracting the average of the signal below the Otsu threshold (Otsu, 1979) from the original image. To estimate the location of the membrane region, we first thresholded the original image using the Otsu algorithm (Otsu, 1979). The mask was then cleaned up using a morphological opening operation. Finally, the eroded mask was subtracted from a dilated version of the mask, selecting a 3D shell around the boundary of the cleaned-up mask. All morphological operations were conducted in three dimensions and with a structuring element of a sphere with radius 2 pixels or 0.42 μm. Within the shell, the average intensity for each channel was measured. For the Dlg and β-Spectrin intensities, Dlg was measured as *HRP-CH 1* and β-Spectrin was measured as *CH 2*. [In the code, to define the membrane, HRP was uses as channel 1 *CH1* and the postsynaptic protein being measured was selected as channel 2 *CH2*.] All measurements were performed in the background-corrected cropped image. The code is available at the following GitHub repository: https://github.com/tp81/neisch-dynein-2021.

### Quantification of glutamate receptor field sizes

Wandering third instar larvae from crosses were dissected and processed as described in the ‘Immunostaining’ section above. Six female animals for each genotype were used. Animals were stained with the same primary and secondary antibody solutions and imaged under the same conditions. Samples were imaged in a Zeiss Cell Observer spinning-disk confocal microscope with a Yokogawa CSU-X1 spinning disk confocal head. Illumination was provided with lasers of 488 and 561 nm and the emitted fluorescence was collected through 503–538 and 580–653 nm emission filters, respectively. Zen2 software, a QuantEM 512SC camera and a 63× Plan Apo 1.4NA objective (0.21 µm pixel size) with a *z*-step size of 0.24 µm were used to acquire the images. NMJ images were acquired at abdominal segments A3 and A4 of muscle 4. To quantify the receptor field size, a script was written in Jython within FIJI. The HRP channel *CH 1* was thresholded using the ‘Moments’ algorithm (Tsai, 1985) to each slice. Each slice of the glutamate receptor subunit channel *CH 2* underwent rolling ball background subtraction with radius 2 pixels (0.42 μm). A 3D mask was created by applying the Bersen local thresholding algorithm (Vatani and Enayatifar, 2015; Sezgin and Sankur, 2004) with radius 10 pixels (2.1 μm) to each slice. All parts in the second mask that were not included in the first were excluded from further analysis. The 3D object counter was used to count and measure all objects with a volume greater than 10 voxels (0.10584 µm^3^). The volume (V) was used to estimate an equivalent sphere radius (r_eq_) by using the following formula: 
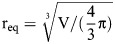
. All code is available on the GitHub repository https://github.com/tp81/neisch-dynein-2021.

### Quantification of BRP puncta per glutamate receptor field

Reconstructed 3D SIM images were analyzed using Imaris 9.5.1 (Bitplane, Belfast, UK). The region of interest containing NMJ4 was first selected. To quantify BRP puncta per image, the BRP channel was defined as spots. The algorithm setting for different spot sizes (region growing) was enabled, as was the shortest distance calculation. The estimated *xy*-diameter was 0.2 µm and the estimated *z*-diameter was 0.8 µm. Background subtraction was enabled. Region growing used local contrast and was manually thresholded. Spots were classified by filtering by quality and manually adjusted for spots visually observed as true signals in the image. To quantify the GluRIIC receptor fields per image, the GluRIIC channel was defined as surfaces. In the algorithm settings, the shortest distance calculation was enabled. Smoothing was applied with a surface detail of 0.065 µm. The background was subtracted with the diameter of the largest sphere that fits into the object, which was 0.244 µm. Thresholding was manually defined to match the visible signal. The setting for split touching objects (region growing) was enabled with a seed point diameter of 0.6 µm. Seed points were classified by a quality filter and manually adjusted. The total number of BRP puncta observed in each NMJ4 image analyzed was divided by the total number of GluRIIC receptor fields observed in the same image to determine the BRP/GluRIIC ratio.

### Electrophysiology

To measure mEJPs, wandering third instar female larvae from crosses of *elav-Gal80/Y; Mef2-Gal4* to *w^1118^* or *UAS-dhc64cRNAi^HMS01587^* were dissected in HL3 saline and visceral organs were removed. Segmental nerves were severed near the ventral nerve cord and the brain was then fully removed. The HL3 saline in the filleted larval preparation was replaced with HL3 saline containing 1.5 mM CaCl_2_ for mEJP recordings. Recordings were performed on muscle 6 of abdominal segments A2 and A3 using sharp glass microelectrodes filled with 3 M KCl. Only recordings with a resting membrane potential of −60 mV or lower were analyzed. Recordings were performed over multiple days. Measurement of mEJPs were performed using Mini Analysis (Synaptosoft, Decatur, GA, USA). The average recording time was 1.8 min for controls and 1.7 min for dynein depletion.

### SIM

Images were acquired in a Nikon Ti-E inverted microscope (Nikon, Melville, NY, USA) equipped with a CFI SR Apo 100× TIRF oil immersion objective lens with NA 1.49 (0.033 µm pixel size). Illumination was provided by 488 and 561 nm lasers fed through a multiple mode fiber to a Nikon N-SIM illuminator with the appropriate diffraction grating. The cognate emission filters used were 522–545 and 605–670 nm, respectively. Images were acquired in 3D SIM mode with an Orca-Flash4.0 V3 sCMOS camera and the SIM image was reconstructed using Nikon software and the default software settings for illumination modulation contrast, high-resolution noise suppression and out-of-focus blur suppression (Nikon Elements 5.11). Pixel size of the reconstructed images was 33 nm.

### β-Spectrin antibody production

A DNA sequence encoding *Drosophila* β-Spectrin amino acids 1013–1463 was PCR amplified from pUASTattB-β-Spectrin (Avery et al., 2017) using the oligonucleotides 5′-GGAATTCCATATGGAGCGCGAAGCCAACAGCATC-3′ and 5′-ACCGCTCGAGCGTCTTCTTCACCACAATCGGTTCG-3′. The PCR product was digested with Nde1 and Xho1 restriction enzymes and inserted into the bacterial expression vector pET-30a(+) (Novagen, Madison, WI, USA) cut with the same enzymes to introduce a His tag at the C-terminus of the β-Spectrin protein fragment. The resulting construct was confirmed by DNA sequencing. The β-Spectrin–His construct was transformed into BL21(DE3) cells (Novagen) and induced for protein expression with 0.5 mM IPTG (Invitrogen). The bacterial pellet was lysed by sonication in binding buffer consisting of PBS with 10 mM imidazole (Sigma-Aldrich) and Complete Protease Inhibitor Cocktail, EDTA-Free (Roche, Mannheim, Germany). After clarification by centrifugation, the lysate was incubated with Ni-NTA agarose resin for binding (Invitrogen) and the resin was then transferred to a Poly-Prep chromatography column (Bio-Rad, Hercules, CA). The β-Spectrin–His protein was eluted in PBS containing 200 mM imidazole and then dialyzed in PBS to remove imidazole. The β-Spectrin–His protein was injected into a guinea pig at Pocono Rabbit Farm and Laboratory (Canadensis, PA, USA), and serum containing the anti-β-Spectrin antibody collected. This antibody is available upon request.

### Dlic-GFP generation

A PCR fragment encoding eGFP was first ligated to the 3′ end of the *dlic* coding sequence. The Dlic-GFP fragment was then fused to ∼2 kb genomic sequences including the 5′ untranslated region and the upstream regulated region used for the *dlic* genomic transgene (Mische et al., 2008). The final construct was cloned into the pCaspeR4 vector (Klemenz et al., 1987) and transgenic flies were generated. The transgene encoding Dlic–GFP is fully functional for rescuing *dlic* lethal mutations to viability. This fly strain is available upon request.

## Supplementary Material



10.1242/joces.263844_sup1 Supplementary information
