# Supplementary material for: Dynein-driven regulation of postsynaptic membrane architecture and synaptic function
Source: J Cell Sci. 2025 Mar 12;138(5):JCS263844. doi: 10.1242/jcs.263844 (PMC11959486; doi:10.1242/jcs.263844)
Supplement: Supplementary information [file joces-138-263844-s1.pdf]

Figure S1

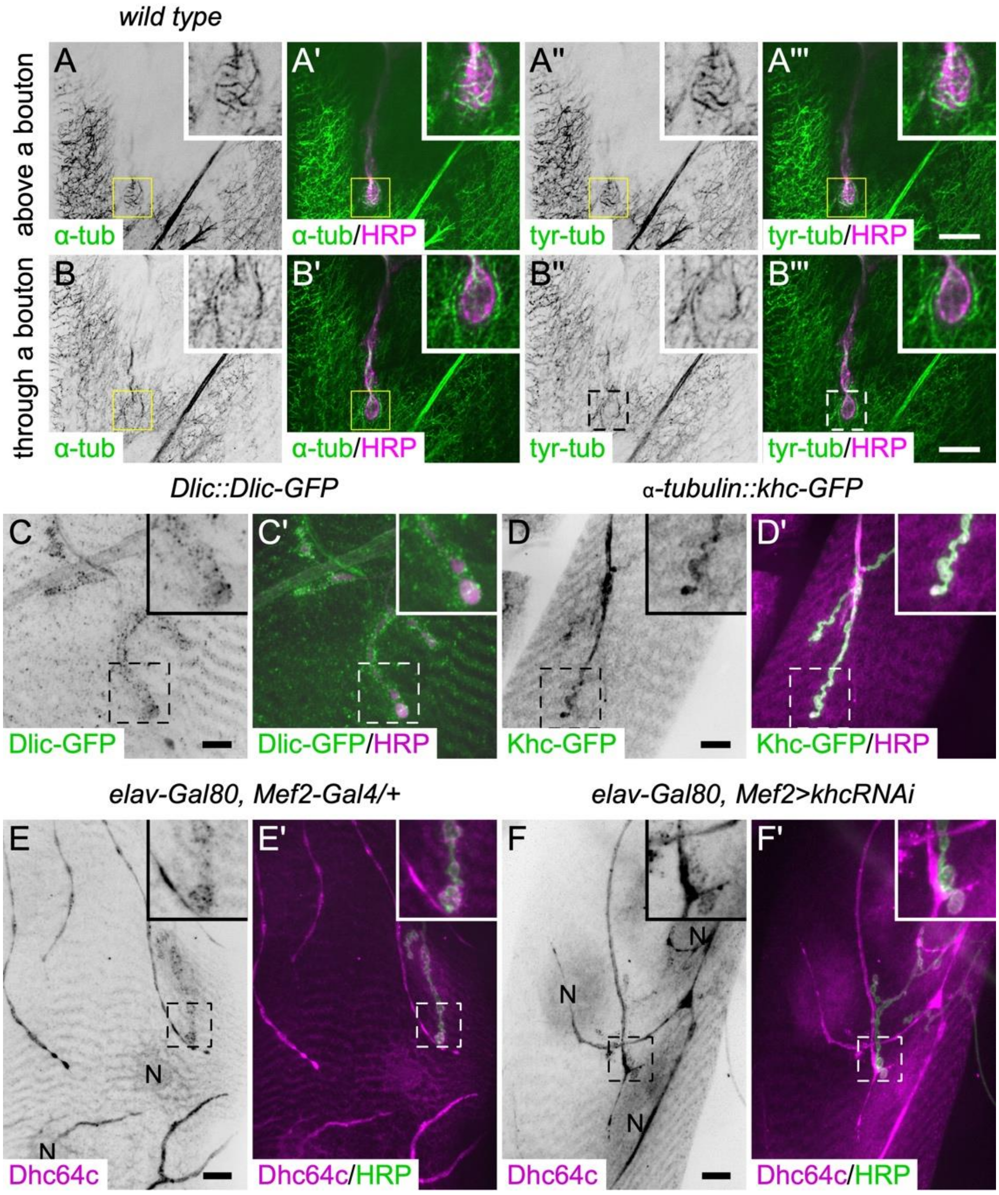

Fig. S1. Dynein's localization at the postsynaptic NMJ is not dependent on Kinesin-1, which predominantly localizes to the presynaptic side of the NMJ.
Microtubules labelled with both pan-α-tubulin and tyrosinated-α-tubulin surround the NMJ on the postsynaptic side. The presynaptic side of the junction is labelled with HRP. A singe section above a bouton **(A-A'''')** and a single section through the center of the same bouton are shown **(B-B'''')**. **C-C')** The dynein light intermediate chain subunit, dlic, tagged with GFP and expressed under its endogenous promoter, has a punctate postsynaptic localization. This localization is similar to that observed for the dynein heavy chain subunit, dhc64c, using an antibody that is specific. **D-D')** Kinesin-1, tagged with GFP and expressed ubiquitously under the α-tubulin promoter, has a strong presynaptic localization at the NMJ, and no specific postsynaptic localization. **E-E')** The localization of dynein at the NMJ in control animals. **F-F')** The localization of dynein at the NMJ is not affected when kinesin-1 is depleted from the muscle by RNAi. Halos of dynein staining are observed around nuclei, labeled with the letter N, when kinesin-1 is depleted from the muscle, which is not observed in controls. Boxed areas are shown as insets. In all images, HRP labels the presynaptic side of the NMJ. Scale, 10μm.

Figure S2

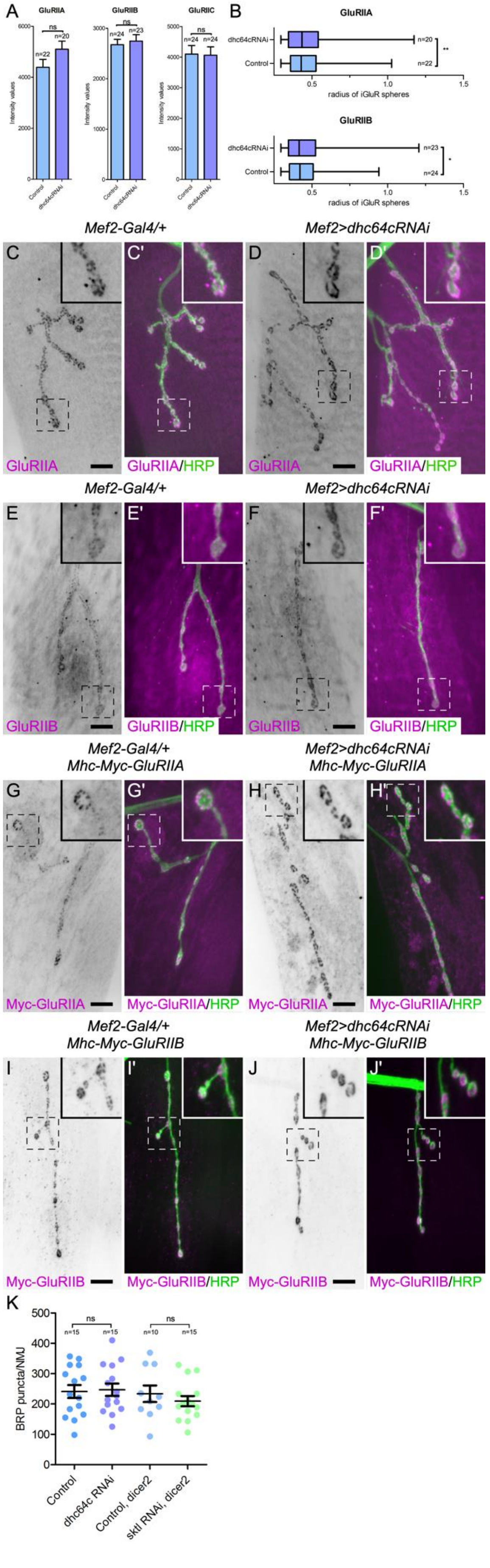

Journal of Cell Science • Supplementary information

Fig. S2. Dynein is required in the muscle for synaptic growth and affects the size of glutamate receptor fields at the NMJ.
A) Intensity values of GluRIIA, GluRIIB, and GluRIIC at the NMJ in control animals, and those depleted of dynein by RNAi. n=number of NMJ analyzed. Unpaired, two-tailed t-test results: ns=not significant. B) The radius of iGluR spheres in control animals, and those depleted of dynein by RNAi. GluRIIA and GluRIIB endogenous antibodies have a high background muscle staining that makes quantification challenging, however a significant change is observed compared to controls. n=number of NMJ analyzed. Unpaired, one-tailed t-test results: **, P<0.005, *, P<0.05. 6 animals for each genotype were analyzed. **C-C')** A representative image of GluRIIA localization at the NMJ is controls and when dynein is depleted in the muscle **(D-D')**. **E-E')** A representative image of GluRIIB localization at the NMJ is controls and when dynein is depleted in the muscle **(F-F')**. **G-G')** Myc-GluRIIA localization at the NMJ in a control animal and in an animal depleted of dynein in the muscle **(H-H')**. **I-I')** Myc-GluRIIB localization at the NMJ in a control animal and in an animal depleted of dynein in the muscle **(J-J')**. **K)** Quantification of the number of presynaptic BRP puncta per neuromuscular junction for controls vs dynein depletion and controls vs Skittles depletion. Each dot is the analysis of a single NMJ. 4 animals were analyzed for all genotypes except the *dicer2* control, for which 3 animals were analyzed. ns=not significant. In all images, HRP labels the presynaptic side of the NMJ. Scale, 10µm.

Journal of Cell Science • Supplementary information

Figure S3

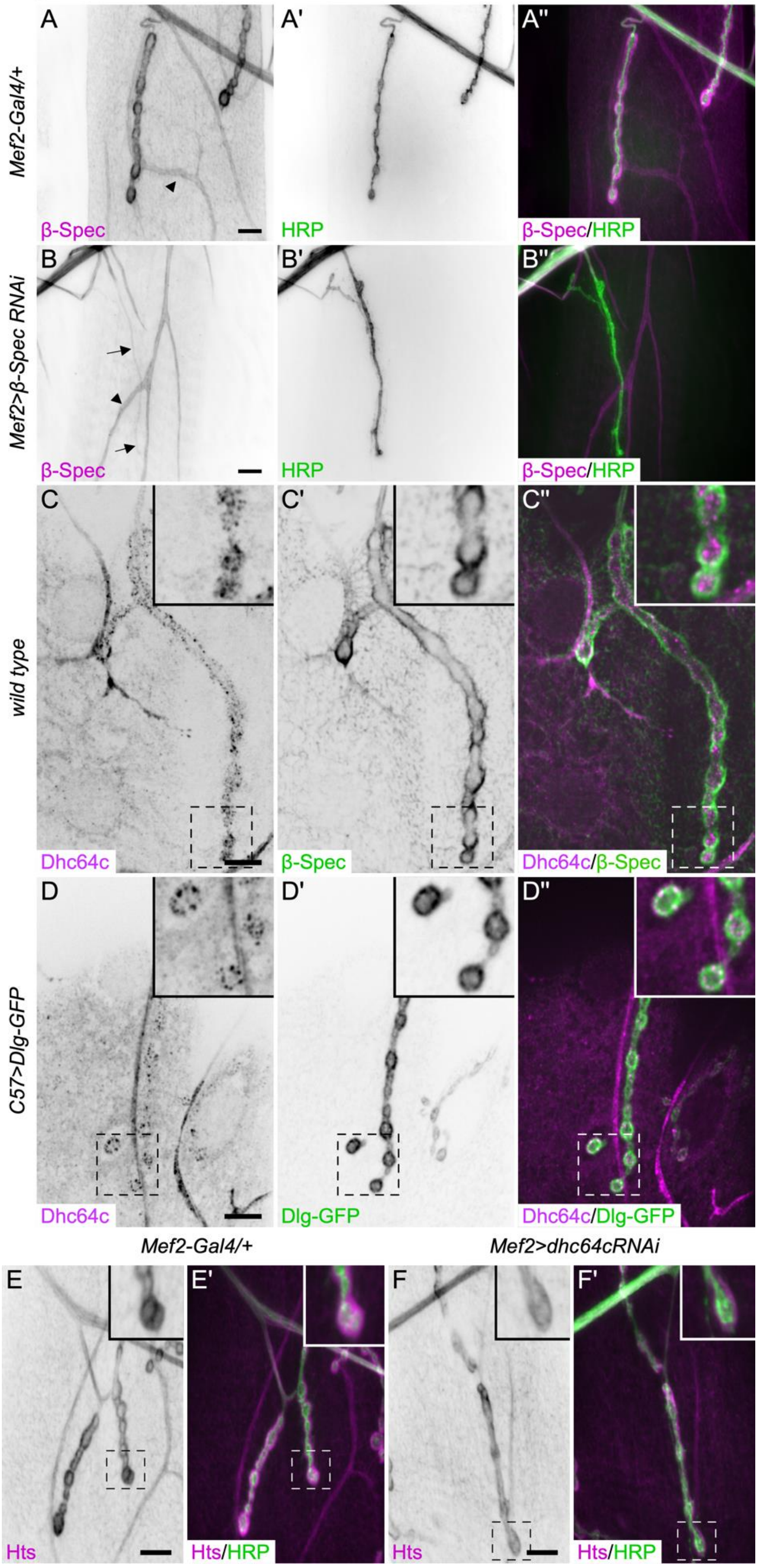

Journal of Cell Science • Supplementary information

Fig. S3. A β-Spectrin antibody shows specificity, postsynaptic Dynein localization overlaps with Dlg, just inside of the β-Spectrin domain, and Dynein depletion reduces Adducin levels.
In order to visualize β-Spectrin at the NMJ, a new antibody was generated. We tested the specificity of this antibody using muscle specific depletion of β-Spectrin. **A-A'')** In control animals β-Spectrin is enriched on the postsynaptic side of the NMJ. **B-B'')** When β-Spectrin is depleted from the muscle, presynaptic β-Spectrin is now able to be visualized with the antibody, as indicated by the arrows. Black arrowheads indicate trachea that stain positive for β-Spectrin. HRP labels the presynaptic side of the NMJ. **C-C'')** Dynein punctae are found inside of the β-Spectrin localization domain at the NMJ. **D-D'')** The localization of the dynein punctae overlaps with Dlg at the NMJ. **E-E')** The localization of Hts (Adducin) at the NMJ in a control animal. **F-F')** The localization of Hts is diminished when dynein is depleted from the muscle. Scale, 10µm.

Figure S4

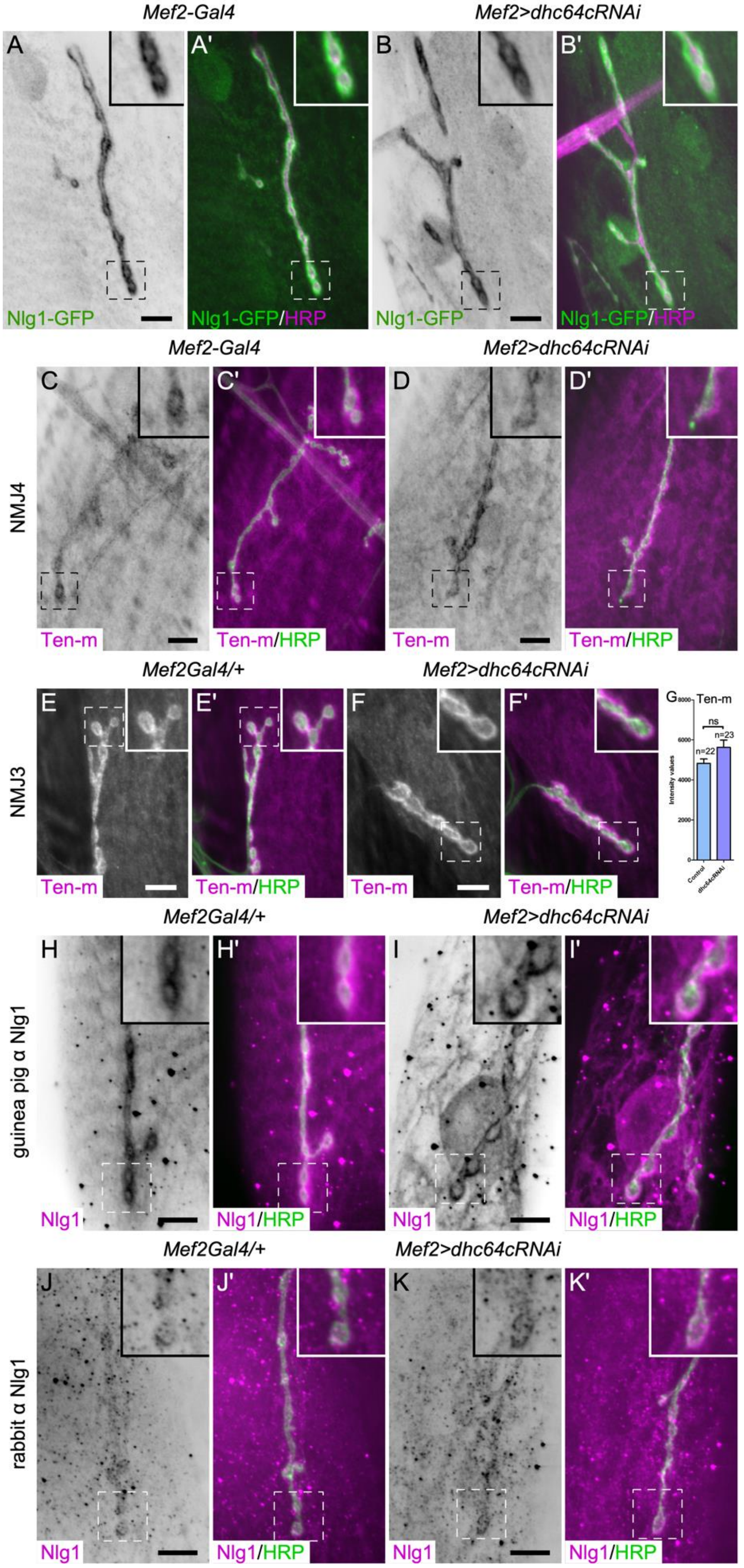
Mef2-Gal4
Mef2>dhc64cRNAi
A
A'
B
B'
Nlg1-GFP
Nlg1-GFP/HRP
Nlg1-GFP
Nlg1-GFP/HRP
Mef2-Gal4
Mef2>dhc64cRNAi
NMJ4
C
C'
D
D'
Ten-m
Ten-m/HRP
Ten-m
Ten-m/HRP
Mef2Gal4/+
Mef2>dhc64cRNAi
NMJ3
E
E'
F
F'
G
Ten-m
ns
n=22
n=23
Intensity values
Control
dhc64cRNAi
Ten-m
Ten-m/HRP
Ten-m
Ten-m/HRP
Mef2Gal4/+
Mef2>dhc64cRNAi
guinea pig α Nlg1
H
H'
I
I'
Nlg1
Nlg1/HRP
Nlg1
Nlg1/HRP
Mef2Gal4/+
Mef2>dhc64cRNAi
rabbit α Nlg1
J
J'
K
K'
Nlg1
Nlg1/HRP
Nlg1
Nlg1/HRP

Fig. S4. Dynein depletion in the muscle does not affect the postsynaptic localization of the transmembrane proteins Ten-m or Nlg1.

A-A') The localization of Nlg1-GFP at the NMJ4 in a control animal. **B-B')** Depletion of dynein from the muscle does not affect the localization of Nlg-GFP at the NMJ. **C-C')** The wild type localization of Ten-m at NMJ4. **D-D')** Depletion of dynein from the muscle does not affect to localization of Ten-m. **E-E')** The localization of Ten-m at NMJ3 of control animals. **F-F')** Ten-m localization at NMJ3 of a postsynaptic dynein depleted animal. **G)** quantification of postsynaptic Ten-m intensity levels in control and dynein depleted animals at NMJ3. n=number of NMJs analyzed. Unpaired two-tailed t-test results: ns=not significant. 6 animals from each genotype were analyzed. **H-H')** The localization of Nlg1 at NMJ4 using a guinea Pig anti-Nlg1 antibody in a control animal and in an animal depleted of postsynaptic dynein **(I-I')**. **J-J')** The localization of Nlg1 at NMJ4 using a rabbit anti-Nlg1 antibody in a control animal and in an animal depleted of postsynaptic dynein **(K-K')**. Boxed areas are shown as insets. In all images, HRP labels the presynaptic side of the NMJ. Scale, 10µm.

Journal of Cell Science • Supplementary information

Figure S5

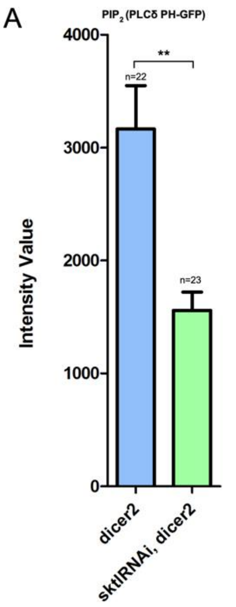

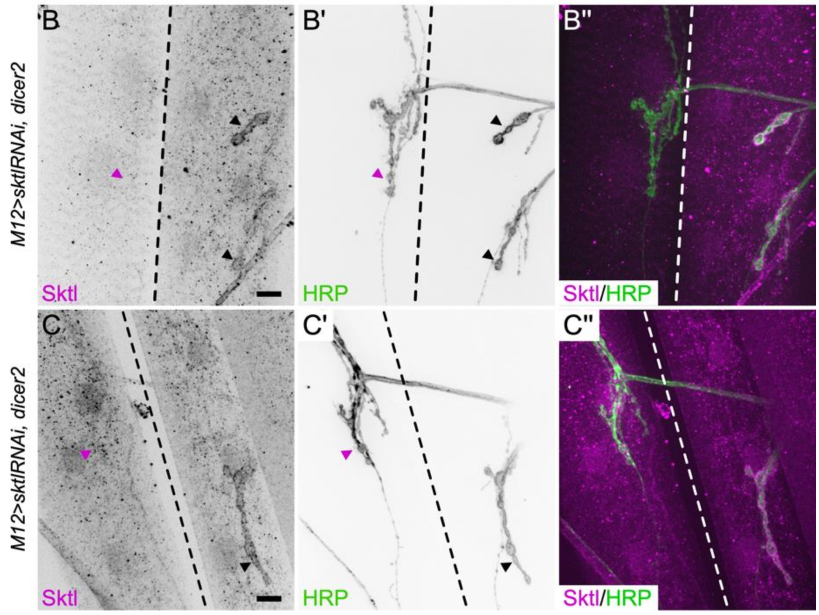

Fig. S5. Sktl can be effectively depleted from the postsynaptic muscle using RNAi. The PIP_2 reporter, PLCδ-PH-GFP, was expressed in the muscle, and levels of PIP_2 at the postsynaptic NMJ membrane were measured for control animals versus those depleted of Skittles **(A).** Skittles depletion significantly reduces the level of PIP_2 at the postsynaptic membrane. **, P<0.005. 4 animals were quantified for *dicer2*, and 6 animals for *sktlRNAi, dicer2*. Sktl was strongly depleted from muscle 12 (left side of the dotted lines), by expressing *sktlRNAi* with the *M12-Gal4* driver **(B, C)**. Red arrowheads indicate the position of muscle 12 NMJs. Muscle 13 (right side of the dotted lines) which does not express *sktlRNAi* is shown for comparison. Black arrowheads indicate the position of muscle 13 NMJs. To visualize Sktl, a Sktl specific antibody was used. HRP labels the presynaptic side of the NMJ **(B', C')**. Scale, 10µm.

Journal of Cell Science • Supplementary information

Figure S6

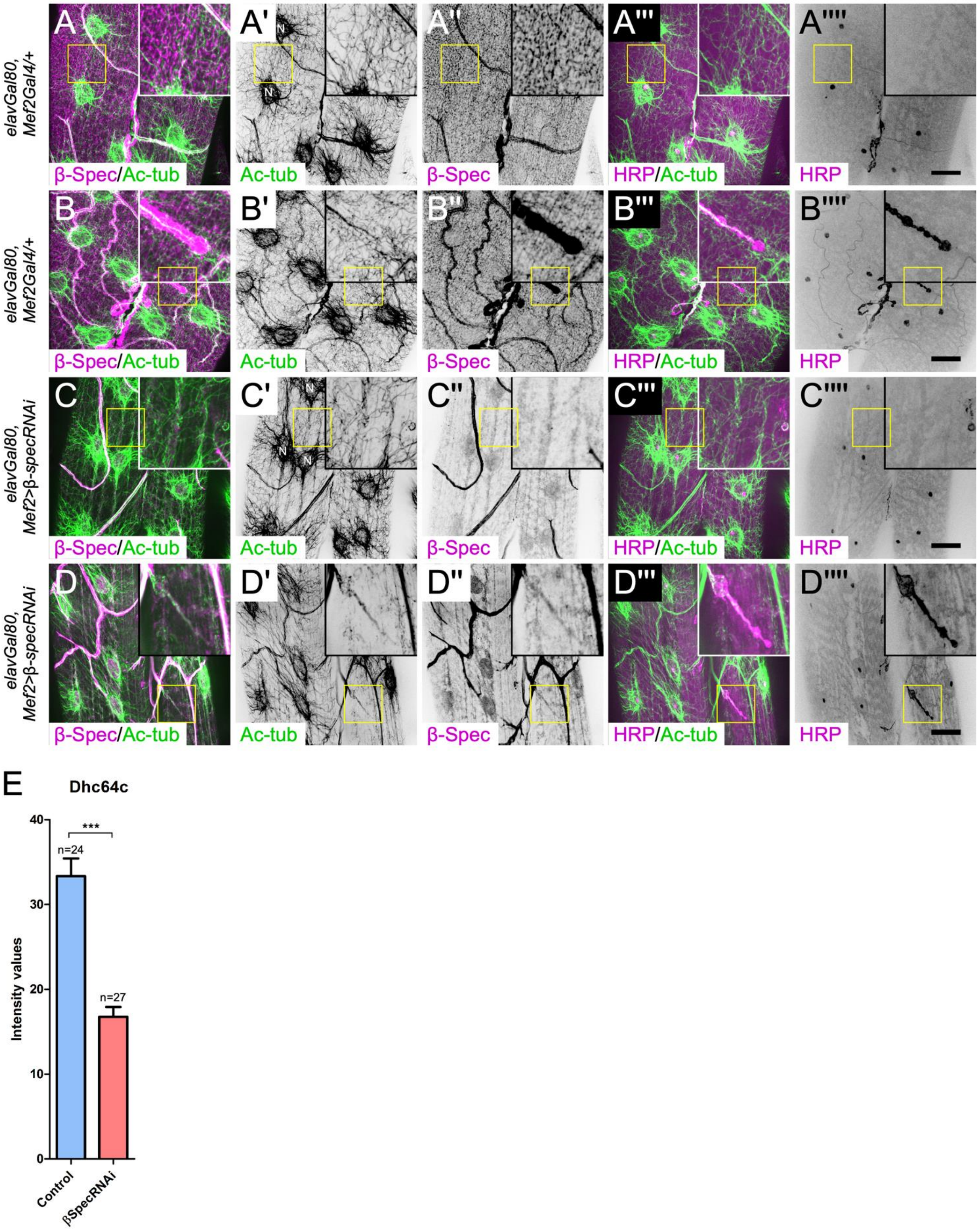

Journal of Cell Science • Supplementary information

Fig. S6. β-Spectrin depletion from the muscle affects the microtubule cytoskeleton.

The underlying spectrin cytoskeleton may organize the microtubule cytoskeleton (A-B’’’). When β-Spectrin is depleted from the muscle the microtubule cytoskeleton appears disorganized (C-D’’’). This is observed for the microtubules between nuclei (A’, C’) and the microtubules surrounding the NMJ (B’, D’). Nuclei in A’, C ’ are labeled with the letter N. Boxed areas are shown as insets. In all images, HRP labels the presynaptic side of the NMJ. Scale, 20μm. Depletion of β-Spectrin from the muscle decreases the amount of postsynaptic dynein at the NMJ (E). Unpaired, two-tailed t-test results: ***, P<0.0001. For the control genotype, 6 animals were analyzed, for the β-Spectrin RNAi genotype, 7 animals were analyzed.

Table S1. Genotypes analyzed and type of image shown in each figure.

Figure #	Genotype	Image Type
1A	w^{1118}	Single section
1B	w^{1118}	Sum of sections
1C	Mef2Gal4/+ (Control)	Max projection of sections
1D	*M12-Gal4, UAS-dhc64cRNAi*^{HMS01587}	Sum of sections
1E	*Dhc64c::Dhc64c*$^{3xHA-WT}$	Sum of sections
1F	*Dhc64c::Dhc64c*$^{3xHA-P1}$	Sum of sections
1G	*M12-Gal4, UAS-khcRNAi*^{GD12278}	Max projection of sections
1H	*M12-Gal4, UAS-khcRNAi*^{GD12278}	Max projection of sections
S1A-B	w^{1118}	Single sections
S1C	*Dlic::Dlic*^{GFP}	Sum of sections
S1D	*α-tubulin::Khc-GFP*	Sum of sections
S1E	*elav-Gal80; Mef2-Gal4/+*	Sum of sections
S1F	*elav-Gal80; Mef2-Gal4, UAS-khcRNAi*^{GD12278}	Sum of sections
2A-B	**Control=** *Mef2-Gal4/+*	
2A-B	**dhc64cRNAi=** *Mef2-Gal4, UAS-dhc64cRNAi*^{HMS01587}	
2A	**elavGal80; dhc64cRNAi=** elav-Gal80; *Mef2-Gal4, UAS-dhc64cRNAi*^{HMS01587}	
S2A-B	**Control=** *Mef2-Gal4/+*	
S2A-B	**dhc64cRNAi=** *Mef2-Gal4, UAS-dhc64cRNAi*^{HMS01587}	
S2C-C'	*Mef2-Gal4/+*	Sum of sections
S2D-D'	*Mef2-Gal4, UAS-dhc64cRNAi*^{HMS01587}	Sum of sections
S2E-E'	*Mef2-Gal4/+*	Sum of sections
S2F-F'	*Mef2-Gal4, UAS-dhc64cRNAi*^{HMS01587}	Sum of sections
S2G-G'	*Mef2-Gal4, Mhc-Myc-GluRIIA*	Sum of sections
S2H-H'	*Mef2-Gal4, Mhc-Myc-GluRIIA, UAS-dhc64cRNAi*^{HMS01587}	Sum of sections
S2I-I'	*Mef2-Gal4, Mhc-Myc-GluRIIB*	Sum of sections
S2J-J'	*Mef2-Gal4, Mhc-Myc-GluRIIB, UAS-dhc64cRNAi*^{HMS01587}	Sum of sections
S2K	**Control=** *Mef2-Gal4/+*	
S2K	**dhc64cRNAi=** *Mef2-Gal4, UAS-dhc64cRNAi*^{HMS01587}	
S2K	**Control dicer2=** elav-Gal80, *Mef2-Gal4, UAS-dicer2*	
S2K	**SktlRNAi dicer2=** elav-Gal80, *Mef2-Gal4, UAS-dicer2, UAS-sktlRNAi*^{JF02796}	
3A-A''		single section
3B-B'	*Mef2-Gal4/+*	single section
3C-C'	*Mef2-Gal4, UAS-dhc64cRNAi*^{HMS01587}	single section
3D	**Control=** *Mef2-Gal4/+*	
3D	**dhc64cRNAi=** *Mef2-Gal4, UAS-dhc64cRNAi*^{HMS01587}	
3D	**Control=** *Mef2-Gal4, Mhc-Myc-GluRIIB*	
3D	**dhc64cRNAi=** *Mef2-Gal4, Mhc-Myc-GluRIIB, UAS-dhc64cRNAi*^{HMS01587}	
3D	**Control=** *Mef2-Gal4, Mhc-Myc-GluRIIA*	
3D	**dhc64cRNAi=** *Mef2-Gal4, Mhc-Myc-GluRIIA, UAS-dhc64cRNAi*^{HMS01587}	
3E-E''	*elav-Gal80, Mef2-Gal4*	Sum of reconstructed images
3F-F''	*elav-Gal80, Mef2-Gal4, UAS-dhc64cRNAi*^{HMS01587}	Sum of reconstructed images
3G	**Control=** *elav-Gal80, Mef2-Gal4*	
3G	**dhc64cRNAi=** *elav-Gal80, Mef2-Gal4, UAS-dhc64cRNAi*^{HMS01587}	
S3A-A''	*Mef2-Gal4/+*	Sum of sections
S3B-B''	*Mef2-Gal4, UAS- βspectrinRNAi*^{GL01174}	Sum of sections
S3C-C''	w^{1118}	Sum of sections
S3D-D''	*C57-Gal4, UAS-Dlg-S97-GFP*	Sum of sections
S3E-E''	*Mef2-Gal4/+*	Sum of sections
S3F-F''	*Mef2-Gal4, UAS-dhc64cRNAi*^{HMS01587}	Sum of sections
4	**Control=** *elavGal80, Mef2-Gal4/+*	
4	**dhc64cRNAi=** *elavGal80, Mef2-Gal4, UAS-dhc64cRNAi*^{HMS01587}	
S4A-A'	*Mef2-Gal4/+*	Sum of sections
S4B-B'	*Mef2-Gal4, UAS-dhc64cRNAi*^{HMS01587}	Sum of sections
S4C-C'	*Mef2-Gal4, UAS-Nlg1-GFP*	Sum of sections
S4D-D'	*Mef2-Gal4, UAS-Nlg1-GFP, UAS-dhc64cRNAi*^{HMS01587}	Sum of sections
S4E-E'	*Mef2-Gal4/+*	Sum of sections
S4F-F'	*Mef2-Gal4, UAS-dhc64cRNAi*^{HMS01587}	Sum of sections
S4G	**Control=** *Mef2-Gal4/+*	
S4G	**dhc64cRNAi=** *Mef2-Gal4, UAS-dhc64cRNAi*^{HMS01587}	
S4H-H'	*Mef2-Gal4/+*	Sum of sections
S4I-I'	*Mef2-Gal4, UAS-dhc64cRNAi*^{HMS01587}	Sum of sections
S4J-J'	*Mef2-Gal4/+*	Sum of sections
S4K-K'	*Mef2-Gal4, UAS-dhc64cRNAi*^{HMS01587}	Sum of sections
5A-A''	*Mef2-Gal4/+* (Control)	Sum of sections
5B-B''	*Mef2-Gal4, UAS-dhc64cRNAi*^{HMS01587}	Sum of sections
5C	*Mef2-Gal4/+*	
5C	*Mef2-Gal4, UAS-dhc64cRNAi*^{HMS01587}	
5C	*G14-Gal4/+*	
5C	*G14-Gal4, UAS-dhc64cRNAi*^{HMS01587}	
5D	*Mef2-Gal4/+*	
5D	*Mef2-Gal4, UAS-dhc64cRNAi*^{HMS01587}	
5D	*Mef2-Gal4, UAS-swRNAi*^{HM05249}	
5D	*G14-Gal4/+*	
5D	*G14-Gal4, UAS-dhc64cRNAi*^{HMS01587}	
5E	*Mef2-Gal4/+*	
5E	*Mef2-Gal4, UAS-dhc64cRNAi*^{HMS01587}	
5F	**Control=** *Mef2-Gal4/+*	
5F	**dhc64cRNAi=** *Mef2-Gal4, UAS-dhc64cRNAi*^{HMS01587}	
5G	**Control=** *Mef2-Gal4, UAS-Nlg1-GFP*	
5G	**dhc64cRNAi=** *Mef2-Gal4, UAS-Nlg1-GFP, UAS-dhc64cRNAi*^{HMS01587}	
5H	**Control=** *Mef2-Gal4/+*	
5H	**dhc64cRNAi=** *Mef2-Gal4, UAS-dhc64cRNAi*^{HMS01587}	
S5A	**dicer2=** *elavGal80, Mef2-Gal4, UAS-PLC δ-PH-EGFP, UAS-dicer2*	
S5A	***sktlRNAi, dicer2=*** *elavGal80, Mef2-Gal4, UAS-PLC δ-PH-EGFP, UAS-sktlRNAi*^{JF02796} *, UAS-dicer2*	
S5B-B''	*M12-Gal4, UAS-sktlRNAi*^{JF02796} *, UAS-dicer2*	Max projection of sections
S5C-C''	*M12-Gal4, UAS-sktlRNAi*^{JF02796} *, UAS-dicer2*	Max projection of sections
6A-A'	*Mef2-Gal4, UAS-PLC δ-PH-EGFP*	Sum of sections
6B'B'	*Mef2-Gal4, UAS-PLC δ-PH-EGFP, UAS-dhc64cRNAi*^{HMS01587}	Sum of sections
6C	**Control=** *Mef2-Gal4, UAS-PLC δ-PH-EGFP*	
6C	**dhc64cRNAi=** *Mef2-Gal4, UAS-PLC δ-PH-EGFP, UAS-dhc64cRNAi*^{HMS01587}	
6D-D'	*elav-Gal80, Mef2-Gal4, UAS-6xMyc-sktl*	Sum of sections
6E-E'	*elav-Gal80, Mef2-Gal4, UAS-6xMyc-sktl*	Max projection of sections
6F-F'	*Mef2-Gal4/+*	Sum of sections
6G-G'	*Mef2-Gal4, UAS-dhc64cRNAi*^{HMS01587}	Sum of sections
6H	**Control=** *Mef2-Gal4/+*	
6H	**dhc64cRNAi=** *Mef2-Gal4, UAS-dhc64cRNAi*^{HMS01587}	
6I-I'	*elav-Gal80, Mef2-Gal4, UAS-dicer2*	Sum of sections
6J-J'	*elav-Gal80, Mef2-Gal4, UAS-dicer2, UAS-sktlRNAi*^{JF02796}	Sum of sections
6K	**Control dicer2=** elav-Gal80, *Mef2-Gal4, UAS-dicer2*	
6K	**SktlRNAi dicer2=** elav-Gal80, *Mef2-Gal4, UAS-dicer2, UAS-sktlRNAi*^{JF02796}	
S6A-A''''	*elav-Gal80, Mef2-Gal4/+*	single section
S6B-B''''	*elav-Gal80, Mef2-Gal4/+*	single section
S6C-C''''	*elav-Gal80, Mef2-Gal4, β-SpecRNAi*^{GL01174}	single section
S6D-D''''	*elav-Gal80, Mef2-Gal4, β-SpecRNAi*^{GL01174}	single section
S6E	**Control =** elav-Gal80, *Mef2-Gal4/+*	
S6E	**β-SpecRNAi=***elav-Gal80, Mef2-Gal4, β-SpecRNAi*^{GL01174}	
7A-A'	*elav-Gal80, Mef2-Gal4, UAS-dicer2*	Sum of reconstructed images
7B-B'	*elav-Gal80, Mef2-Gal4, UAS-dicer2, UAS-sktlRNAi*^{JF02796}	Sum of reconstructed images
7C	**Control=** *elav-Gal80, Mef2-Gal4*	Data taken from 3G for comparison
7C	**dhc64cRNAi=** *elav-Gal80, Mef2-Gal4, UAS-dhc64cRNAi*^{HMS01587}	Data taken from 3G for comparison
7C	**Control dicer2=** elav-Gal80, *Mef2-Gal4, UAS-dicer2*	
7C	**SktlRNAi dicer2=** elav-Gal80, *Mef2-Gal4, UAS-dicer2, UAS-sktlRNAi*^{JF02796}	
7D-D'	w^{1118}	Sum of reconstructed images
7E-E'	w^{1118}	Sum of reconstructed images

Journal of Cell Science • Supplementary information
