# Supplementary material for: Dynein-driven regulation of postsynaptic membrane architecture and synaptic function
Source: J Cell Sci. 2025 Mar 12;138(5):JCS263844. doi: 10.1242/jcs.263844 (PMC11959486; doi:10.1242/jcs.263844)
Supplement: Supplementary file 1 [file joces-138-263844_review_history.pdf]

Journal of
Cell Science

Dynein-driven regulation of postsynaptic membrane architecture and synaptic function

Amanda L. Neisch, Thomas Pengo, Adam W. Avery, Min-Gang Li, Thomas S. Hays
DOI: 10.1242/jcs.263844

Editor: Giampietro Schiavo

Review timeline
Original submission: 27 September 2021
Editorial decision: 15 November 2021
Resubmission: 13 January 2025
Accepted: 18 January 2025

Original submission

First decision letter

MS ID#: JOCES/2021/259416

MS TITLE: Dynein acts to cluster glutamate receptors and traffic the PIP5 kinase, Skittles, to regulate postsynaptic membrane organization at the neuromuscular junction

AUTHORS: Amanda L. Neisch, Thomas Pengo, Adam W. Avery, Min-Gang Li, and Thomas S. Hays

We have now reached a decision on the above manuscript.

To see the reviewers' reports and a copy of this decision letter, please go to: https://submit-jcs.biologists.org and click on the 'Manuscripts with Decisions' queue in the Author Area. (Corresponding author only has access to reviews.)

As you will see, the reviewers raise a number of substantial criticisms that prevent me from accepting the paper at this stage. They suggest, however, that a revised version might prove acceptable, if you can address their concerns. If you think that you can deal satisfactorily with the criticisms on revision, I would be pleased to see a revised manuscript. We would then return it to the reviewers.

We are aware that you may be experiencing disruption to the normal running of your lab that makes experimental revisions challenging. If it would be helpful, we encourage you to contact us to discuss your revision in greater detail. Please send us a point-by-point response indicating where you are able to address concerns raised (either experimentally or by changes to the text) and where you will not be able to do so within the normal timeframe of a revision. We will then provide further guidance. Please also note that we are happy to extend revision timeframes as necessary.

Reviewer 1 Advance Summary and Potential Significance to Field:
This manuscript describes for the first time the localization of dynein at the post-synaptic side of the neuromuscular junction (NMJ) in Drosophila larvae, and provides novel insight into its function at this site. The authors observe dynein clusters on the post-synaptic side of type I glutamatergic type I synaptic terminals, where it overlaps to a large extent with glutamate receptors, and is sometimes seen by SIM as a spot within a ring of receptors. By depleting dynein heavy chain (dhc) expression by RNAi specifically in some muscle cells but not others, they find that loss of dhc leads to a reorganization of GluRII clusters into patches, and to a reduced number of synaptic boutons without affecting muscle area. This has functional consequences, leading to an increase in

© 2025. Published by The Company of Biologists under the terms of the Creative Commons Attribution License (https://creativecommons.org/licenses/by/4.0/).

amplitude and frequency of spontaneous synaptic transmissions. The authors speculate that dynein is physically tethering glutamate receptors at the membrane, but there is no evidence to support this conclusion, and the reason for the altered distribution is not identified. In addition to these changes in glutamate receptor localization and synaptic function, the authors find that dhc depletion leads to a great reduction in spectrin labelling in the post-synaptic region, and that of the spectrin interactor adducin, while the post-synaptic membrane associated protein discs large (Dlg) and adhesion proteins Ten-m and Nlg1 are not affected. This reduction in spectrin and adducin, both of which bind PIP2, correlates with a decreased PIP2 level at the post-synaptic membrane, and in the amount of the phosphoinositol 4-phosphate 5-kinase, Skittles, found in boutons. The loss of Skittles, leading to reduction in PIP2, spectrin and adducin, does not cause changes in GluRII localization, however, suggesting that dynein affects two independent pathways post-synaptically. These are all interesting observations, but while the authors speculate about what dynein's role is, explanations for the phenotypes are not yet there. Some simple additional experiments would help test some of the suggested mechanisms, as described below.

Reviewer 1 Comments for the Author:

Major points:
1. One potential role for dynein suggested by the authors is that it anchors microtubule plus ends at the plasma membrane, and that this somehow affects GluRII organization. However, the only data on MT localization presented is labelling with anti-acetylated tubulin, which only recognises a subset of MTs that run in parallel to the membrane. The use of anti-tyrosinated tubulin would reveal dynamic MTs, and labelling with antibodies to a +TIP protein would show if plus ends are indeed anchored at dynein spots. The labelling should also be performed under knock-down conditions as well. Obviously, live cell imaging of +TIP growth would be best, but this would be outside the scope of this work.
2. Identifying the orientation of MTs may also shed light on a contradiction to the +end anchoring hypothesis, which is that an inactive dhc mutant does not localize to boutons like wild type, which suggests it needs to move towards MT minus ends to reach the synapse. The same would be true for Skittles, which the authors claim is a dynein cargo transported to the post-synaptic NMJ (although that is not shown directly).
3. Another notable omission is any consideration of changes in actin localisation following the loss of spectrin from post-synaptic regions. This should be assessed in the various knock-down conditions.
4. The localization of dynein and GluRII is intriguing. Looking at the images, is it possible that the colocalization is more pronounced at the terminal bouton? The authors also point out that it resembles that of the active zone labelled by BRP. Is it possible to perform dual labelling to test this? How common is this localization?
5. The authors mention that NCAM (Fas2 in Drosophila) could provide another route for dynein recruitment to the membrane. Can this be tested straightforwardly?
6. Some of the data derived after depletion of Skittles are problematic. Firstly, it seems that there is a problem with the Skittles depletion that is explained on p9, while this method is first described on p8. This depletion is reported to "increase overall GluRII levels at the membrane"□ and to increase "cytoplasmic GluRIIC signal throughout the muscle"□. Given that this is an integral membrane protein, the latter conclusion doesn't make sense. Also, the background level is very different between fig 8A and B., whereas the opposite is true for 8D and 8E. Can technical issues be ruled out? Does the increased background provide a simple explanation for the higher signal at the NMJ in these samples? The SIM in 8G and H is of lower quality compared to that in fig. 3.
7. Previous work (Eaton et al., 2002) investigated the role of dynactin in NMJs, and found no post-synaptic role. How do the authors reconcile this with their results? This should at least be discussed.

Minor points:
1. Please explain how 'HRP' labels neurons (nothing is included in the methods). Is this short-hand for anti-HRP labelling? If so, include appropriate citations for why this works.
2. Please explain for the non-expert how different synapse types are identified in this system.
3. Does depletion of khc lead to a more disorganized dhc pattern (Fig. 1F)?
4. Figure S2A contains data from Fig. 2. I would suggest putting all the data in fig. 2. The significance of the elavGal80 should be discussed at this point (p4), rather than later (p5).

© 2025. Published by The Company of Biologists under the terms of the Creative Commons Attribution License (https://creativecommons.org/licenses/by/4.0/).

5. The method for analysing GluRII distribution is hard to understand. How reliable is the method, which seems to calculate spheres, whereas the label is clearly in patches? Perhaps a worked example in supplemental material would help. In the analysis of BRP puncta distribution, does the total number of BRP spots change, or their intensity? How is a GluRII spot defined in these images? Many look like stripes.
6. All the figures include red-green merges, although b+W images are also provided. The authors should consider using green and magenta for the benefit of colour-blind readers.
7. The manuscript would benefit from some changes to the writing style. The present tense is used too much to describe results. There is also repetition of results text in the figure legends, and little description of the actual experimental detail.
8. The figure legends provide numbers of NMJs analysed, and the methods state the number of animals used, but it would be better to have all of that information in the legends (e.g. 20 NMJs from 6 animals).

*****
Reviewer 2 Advance Summary and Potential Significance to Field:
The manuscript entitled 'Dynein acts to cluster glutamate receptors and traffic the PIP5 kinase, Skittles, to regulate postsynaptic membrane organization at the neuromuscular junction'¤ explore the involvement of the microtubule (MT) motor dynein on the postsynaptic side of neuronal connections. Transport with MTs and the associated motors are less well known on the postsynaptic side than on the presynaptic side of neuronal connections.
In order to analysis this aspect the authors have focus their study on the Drosophila Type 1 neuromuscular junction (NMJ) synapses which can be considered functionally equivalent to mammalian postsynaptic dendritic spines.
The authors find that dynein presents a postsynaptic localization at NMJs that has not been previously reported. Consistent with this, they noticed that stable MT although faintly overlap with b-spectrin at the NMJ. It would have been relevant to also investigated non-ac MTs.
They show that this dynein localization occurs only in Type 1 extremity but not in type II or III. They convincingly show the specificity of dynein localization at NMJ by RNAi directed against dhc 64c in a muscle subset.
The Kinesin-1 and dynein motors are known to interact and function in a cooperative mode in particular for nuclear positioning in muscle cells. Possibly suggesting that the distribution of dynein at the NMJ is related to kinesin-1 activity. The authors clarify this point by showing that Kinesin-1 is not located at NMJ and that Kinesin-1 depletion does not impact dynein localization at NMJ. They further show that dynein localization at NMJ is connected to its motor activity.
The authors next investigate the dynein requirement at NMJ. They first show that post synaptic dynein is necessary for synaptic growth without affecting muscle area and second provide evidences that dynein is required for the proper receptor clustering but not their localization toward the NMJs. They further show that postsynaptic dynein functions in modulating synaptic transmission.
The authors further address how dynein functions to impact NMJ organization. They identify that dynein is required for the postsynaptic localization at NMJs of b-spectrin and its associated protein aducin/Hts. They identify that dynein is needed for PIP2 production at the NMJ, by transporting the PIP5Kinase sktl. There results further indicate that the transport of sktl by dynein to the NMJ is necessary for the organization of the spectrin cytoskeleton but that dynein functions largely independent of sktl for glutamate receptor clustering.

Overall the manuscript provides several interesting and new findings concerning postsynaptic dynein requirement at the NMJ.

The quality of the results presented is very good in general. The figures are generally of good quality and well explained. I consider that this manuscript deserved publication in Journal of Cell Science, I would nonetheless recommend some minor revisions.

Reviewer 2 Comments for the Author:
1: The authors analyzed the distribution of acMT at the NMJ. They faintly overlap with b-spectrin. What is the distribution of non-acetylated MTs at NMJs.

2: The description concerning the figure 3 E-G in the result section is difficult to follow. It is not clear from the text that bruchpilot is located on the presynaptic side. It is not clear neither what we learn by following the brp/receptor ratio . From the Sim analysis, in control, brp and GluRIIC

© 2025. Published by The Company of Biologists under the terms of the Creative Commons Attribution License (https://creativecommons.org/licenses/by/4.0/).

partially overlap . In absence of dynein, brp appears inside the cluster but excluded from GluRIIC signal.

*****

Reviewer 3 Advance Summary and Potential Significance to Field:
In the paper Neisch and colleagues address the role of postsynaptic Dynein at the larval neuromuscular junction (NMJ) of Drosophila. Interestingly, Dynein heavy chain (Dhc) is present in a punctate pattern surrounding, and potentially associated with, postsynaptic glutamate receptor clusters (GluRs).
Using targeted postsynaptic RNAi the authors demonstrate that absence of Dhc64c results in an increase in postsynaptic GluR cluster sizes that are now opposite by a larger number of presynaptic active zone sites which is nicely demonstrated by SIM microscopy. Likely as a direct consequence of this increase in cluster size the amplitude of miniature release events is increased in these animals. To address the cellular mechanisms by which Dhc64c affects GluR cluster organization the authors test different known postsynaptic components that have previously been implicated in GluR cluster organization. They demonstrate that postsynaptic knock down of Dhc64c strongly reduced postsynaptic b-spectrin, a-spectrin and hts, a complex that has been identified (at least a- and b-spectrin) as essential for the regulation of GluR clustering and miniature amplitude control. As postsynaptic levels of PIP2 and of the PIP2 producing kinase Skittles are also reduced at the postsynaptic SSR in the absence of Dhc64c they propose that Dynein localizes Skittles to the postsynaptic membrane to generate PIP2 to organize the postsynaptic spectrin-associated cytoskeleton. This is again based on a prior publication demonstrating direct interaction between PIP2 and Hts at the postsynaptic membrane (Wang et al., 2014). However, as knock down of Skittles only mildly affects GluR clustering despite a reduction in postsynaptic beta-spectrin they propose an additional direct anchoring function of Dynein for GluRs. This is based on an occasional localization to the center of GluR clusters.

While the authors make several interesting observations that link postsynaptic Dynein to a) the organization of postsynaptic lipid kinases and lipid metabolism, b) the organization of the postsynaptic spectrin cytoskeleton and c) GluR cluster organization and function, the manuscript currently fails to provide enough mechanistic data to support their strong conclusions.

Reviewer 3 Comments for the Author:
General criticism:
The authors convincingly demonstrate that a knock down of postsynaptic Dynein severely impairs the cytoskeletal organization of the postsynaptic SSR. The observed increase in GluR cluster size and the corresponding increase in miniature amplitudes would be entirely consistent with the previously reported requirement of postsynaptic beta-spectrin for these processes (Pielage et al., 2006). In principle, also their model that PIP2 (potentially generated by Skittles at the postsynaptic membrane) regulates beta-spectrin complex assembly is supported by the nice demonstration of a direct interaction between PIP2 and Hts published in Wang et al., 2014.
However, as the authors only correlate the phenotypes of different knock down situations that all result in very variable effects of potential target genes it is currently not possible to deduce meaningful mechanistic insights from this work. The authors should complement their approaches with targeted point mutations or rescues (or at least overexpression) of mutant isoforms lacking interaction domains to determine cause and consequence.
Currently, very strong conclusions are made that are not supported and the authors fail to address alternative possibilities that could equally explain the data. As an example, the impairment of postsynaptic beta-spectrin localization may be responsible for the observed reduction in Hts level. Despite the potential “upstream”¤ position of the adaptor protein Ankyrin as an interactor of CAMs, its postsynaptic localization entirely depends on beta-spectrin.
Similarly, it is not consistent that e.g., Dlg level or distribution are not at all affected in the Dhc knock down, if beta-spectrin and Hts levels are indeed reduced to less than 30%. It has been reported that loss of Hts and beta-spectrin impair Dlg level (Hts) and localization (beta-spectrin) (again, addressed in Pielage et al., 2006 and Wang et al., 2014).
Loss of Dynein clearly disrupts postsynaptic organization but the mechanistic reasons for this are still unclear, and the claims of the authors are currently insufficiently supported.

Specific points:

© 2025. Published by The Company of Biologists under the terms of the Creative Commons Attribution License (https://creativecommons.org/licenses/by/4.0/).

1) The authors claim that the localization of Dynein to the postsynaptic SSR depends on its motor function and thus on a MT based transport. However, while the authors show in Figure 1 that tubulin is present in the vicinity of postsynaptic SSR (marked by b-spectrin), the MT organization does not support a potential targeting of Dynein or Dynein-cargo to GluR clusters. Currently only maximum projections of 3D image stacks are shown that do not allow a 3D reconstruction of MT localization. The highlighted MTs may be below the synaptic bouton. Single sections or a 3D SIM reconstruction would be necessary to demonstrate that MTs reach GluR clusters to support the proposed MT-dependent Dynein localization at the GluRs that would be necessary for e.g., the transport of Skittles.

2) Similarly, the claim that Dynein localizes to the center of GluR clusters should be supported by 3D reconstructions of the SIM images. In addition, the data should be quantified. From the images presented in Figure 3A′′ and Figure 8G´, H′only very few Dynein punctae localize to GluR clusters – others are at a clear distance or next to GluR clusters. 3D data and quantifications are necessary to support this claim.
Do the authors observe a similar localization for Dlic-GFP? – This is not clear from Figure S1.

4) All GluR phenotypes are consistent with the absence of beta-spectrin. However, the absence does not seem to be 74% at all NMJs. Such a decrease should result in a severe disruption of Dlg (see above). In Figure 5D still significant levels of beta-spectrin are evident at the NMJ in the background (partially below the inset). The authors should plot individual data points to allow a better judgement of variability (this applies to all data sets presented)

5) The authors propose a linear sequence from Skittles via PIP2-Hts to beta-spectrin. Why are PIP2 and Hts level much less affected than beta-spectrin levels in the absence of Dhc64c? Can the small reductions in PIP2 level indeed explain the dramatic loss of beta-spectrin?

6) The authors claim on page 8: “Together these results suggest that dynein is required to transport Skittles to the NMJ and that Skittles localization is required to locally produce postsynaptic PIP2.” However, they do not provide any data supporting these claims. Why do the authors omit an analysis of PIP2 abundance in Skittles RNAi or overexpression animals? Does it indeed locally regulate PIP2?
What happens to Hts localization after Skittles knock down? Do levels go down in correlation with a reduction in PIP2 and does this sufficiently explains the observed reduction in beta-spectrin? The image in Fig. 7 J still shows much higher beta-spectrin level than observed after the knock down of Dhc64c and not like a 62% knock down. Again, the level of beta-spectrin seem to directly correlate with GluR clustering.

7) The observed effects on GluRIIC levels in the Skittles are not supported by the data. In the SIM analysis in Fig. 8E there does not seem to be an effect on either GluRIIC abundance or changes in membrane/ extrasynaptic GluRIIC levels. The reported defect in Fig. 8 B, C is simply not evident in the higher quality SIM analysis.

8) The authors cannot claim that Dynein acts directly to position GluR clusters only on co-localization data – how is this supposed to work. Does it bind directly to GluRs – this should then be demonstrated biochemically.

Minor points
1) The authors demonstrate that GluRII cluster size is increased in the absence of Dhc64c. The current blotting of the data shows the size increase for GluRIICc

---

Resubmission

MS ID#: jcs.263844

MS TITLE: Dynein-Driven Regulation of Postsynaptic Membrane Architecture and Synaptic Function

© 2025. Published by The Company of Biologists under the terms of the Creative Commons Attribution License (https://creativecommons.org/licenses/by/4.0/).

AUTHORS: Amanda L. Neisch, Adam W. Avery, Mingang Li, Thomas Pengo, Thomas S. Hays

Author response to reviewers' comments

We appreciate the comments and feedback of the reviewers on how to strengthen our manuscript to further support the data we presented. We wish to emphasize that the main point of this paper was the novel finding that the dynein motor complex had a role on the postsynaptic side of glutamatergic NMJ that influenced both the structural organization, including the localization of Skittles/PIP2, and the physiology as measured by mEJPs. We have re-written and removed text to focus on the key findings, added new figures to address reviewers' comments, and have moved some of the data that we felt did not address the main point of the paper to supplemental figures. Text highlighted in yellow has been added to address reviewer's comments. A point-by-point response to the reviewers' comments are below.

Figure changes are noted below:
Figure 1- Panels E, F were previously G, H. Panels G,H were previously E,F.
Figure 2- Panels A, A' were replaced with Panels from S2 A, A'.
Figure 3-From panel D, GluRIIA and GluRIIB were moved to Figure S5.
Figure 5-Panels B and C were moved to Figure S3 C, D. Previous Figure 6A, B, C are now panels F, G, H
Figure 6- This was previously Figure 7.
Figure 7-This was previously Figure 8. A-C have been removed. D-I were renamed A-F.
Figure S1-Panels A-B''' are new data. Previous panels A-D were renamed C-F.
Figure S2- Previous panels A, A' were moved into Figure 2. Previous panel B is now A. Panel B is data Figure 3D. Panel K is new data.
Figure S3- Panels C, D are from previous Figures 5B, C. Panels E, F are from previous Figures 6D, E.
Figure S5- Panel A is new data. Previous panels A and B are now C-D. Previous panels C-D were removed.
Figure S6- This figure is new data.

Reviewer 1 Comments for the Author:
Major points:
1. One potential role for dynein suggested by the authors is that it anchors microtubule plus ends at the plasma membrane, and that this somehow affects GluRII organization. However, the only data on MT localization presented is labelling with anti-acetylated tubulin, which only recognises a subset of MTs that run in parallel to the membrane. The use of anti-tyrosinated tubulin would reveal dynamic MTs, and labelling with antibodies to a +TIP protein would show if plus ends are indeed anchored at dynein spots. The labelling should also be performed under knock-down conditions as well. Obviously, live cell imaging of +TIP growth would be best, but this would be outside the scope of this work.

We agree that we have not extensively characterized postsynaptic microtubule localization, but did not intend this as a major point in the paper. We were speculating on how dynein loss of function might account for the observed impact on glutamate receptor organization. That said, we have now examined the tyrosinated microtubules as suggested by the reviewer 1, as well as the distribution of microtubules revealed using a pan alpha-tubulin antibody. We have included this data in the Figure S1A-B''''. Both tyrosinated and acetylated microtubules surround the NMJ.

We also looked at overexpression of EB-1-GFP, a +TIP protein, to identify the plus-ends of microtubules as suggested. Unfortunately, the cytoplasmic levels of EB-1-GFP were too high to distinguish plus-ends. We further characterized EB-1 localization using an EB-1 specific antibody. Although we see some postsynaptic NMJ signal with this antibody, the observations are not definitive. EB-1 only binds to the growing end of microtubules. If MT plus-ends are bound by dynein and being pulled on, it is unclear if these MTs would be growing and bind EB-1.

We also attempted to visualize the minus-ends of microtubules using overexpression of CAMSAP-GFP, but this reporter decorated much more than just the ends of microtubules when expressed in the muscle. Moreover, overexpression of this construct appeared to disrupt the normal organization of the microtubule cytoskeleton. Finally, to address microtubule organization we looked at a Patronin-YFP endogenous tagged protein, but the signal was not strong enough in

© 2025. Published by The Company of Biologists under the terms of the Creative Commons Attribution License (https://creativecommons.org/licenses/by/4.0/).

muscle tissue after fixation and antibody staining. Fixation is necessary, so that we can counterstain with HRP antibody to distinguish the presynaptic from the postsynaptic signal, and the position of the NMJ.

2. Identifying the orientation of MTs may also shed light on a contradiction to the +end anchoring hypothesis, which is that an inactive dhc mutant does not localize to boutons like wild type, which suggests it needs to move towards MT minus ends to reach the synapse. The same would be true for Skittles, which the authors claim is a dynein cargo transported to the post-synaptic NMJ (although that is not shown directly).

We tried to identify the orientation of the microtubules, as mentioned above. A simple explanation could be that microtubules are oriented in both directions. It is also possible that dynein may be transported to the NMJ and then be tethered at the membrane. Dynein's motor activity is somehow needed to get to the NMJ, as we have shown (Former Figure 1H, now figure 1F). The original text surrounding Figure 1F says that the data suggests at least some of the microtubules are oriented with their minus-ends toward the synaptic terminal.

3. Another notable omission is any consideration of changes in actin localisation following the loss of spectrin from post-synaptic regions. This should be assessed in the various knock-down conditions.

The reviewer will know that this is very hard to do because of the close distance between the synaptic terminal and the underlying muscle fibers which contribute an overwhelming signal when monitoring F-actin localization. The only terminals people have been able to look at are in muscle 12, which are not the muscles that we have used for all our other analyses. As such, we do not make any specific conclusion about the actin cytoskeleton itself in our manuscript.

4. The localization of dynein and GluRII is intriguing. Looking at the images, is it possible that the colocalization is more pronounced at the terminal bouton? The authors also point out that it resembles that of the active zone labelled by BRP. Is it possible to perform dual labelling to test this? How common is this localization?

The colocalization of dynein and GluRII is not limited to the terminal bouton. Th section shown is a single plane through the terminal bouton, but there are other boutons where the overlap is good as well. The magnified SIM images presented are not taken from the terminal boutons (now Figures 7D-E). Both the BRP and Dynein antibody are mouse monoclonal antibodies, and although their localization is similarly at the center of GluR clusters, they are on opposite sides of the junction so looking at their co- localization would ask a different question, not addressed in our manuscript.

5. The authors mention that NCAM (Fas2 in Drosophila) could provide another route for dynein recruitment to the membrane. Can this be tested straightforwardly?

We tried Fas2 RNAi but animals are not viable at 29 degrees and at a lower temperature the animals remain tiny never reaching the size of a 3rd instar larvae. We dissected these animals and the glutamate receptors appear larger but because of they appear developmentally delayed, we do not want to make any specific conclusions.

6. Some of the data derived after depletion of Skittles are problematic. Firstly, it seems that there is a problem with the Skittles depletion that is explained on p9, while this method is first described on p8. This depletion is reported to "increase overall GluRII levels at the membrane" and to the increase "cytoplasmic GluRIIC signal throughout the muscle". Given that this is an integral membrane protein, the latter conclusion doesn't make sense. Also, the background level is very different between fig 8A and B., whereas the opposite is true for 8D and 8E. Can technical issues be ruled out? Does the increased background provide a simple explanation for the higher signal at the NMJ in these samples? The SIM in 8G and H is of lower quality compared to that in fig. 3

The background does not account for the difference in higher signal, background is subtracted out in the calculation. We agree that we do not understand the increase in cytoplasmic GluRIIC, but

© 2025. Published by The Company of Biologists under the terms of the Creative Commons Attribution License (https://creativecommons.org/licenses/by/4.0/).

there are less punctae. Previous Fig 8A and 8B were confocal images shown under the exact same settings, whereas 8D and E were SIM images (which are widefield, not confocal) that have been 3D reconstituted. When the SIM images were adjusted, the settings were not kept the same as the important point here was the receptor field size using 3D reconstruction SUM images. These images are therefore not directly comparable.

In reviewing the data to address the reviewer's question, we find that we do not have a way to determine if the puncta throughout the muscle are true glutamate receptor containing puncta or antibody background/debris. While others have published (Deivasigamani et al, 2015-PMID: 26290519, Kim et al., 2015-PMID: 25723514) that these puncta are extrasynaptic GluRII receptors that can change distribution in different genetic backgrounds, we think it best to remove the text that talks about the extrasynaptic vesicles as this is a minor point in the paper. Former Figures 8A, B were removed, and 8D,E are now 7A,B.

The SIM images in 8G and H (Now 7D and E) that the reviewer references are images of dynein localization in GluRIIC vs the localization of BRP and GluRIIC in Figure 3. The antibodies and proteins recognized are different, so these images are not comparable.

7. Previous work (Eaton et al., 2002) investigated the role of dynactin in NMJs, and found no post-synaptic role. How do the authors reconcile this with their results? This should at least be discussed.

We have added in text to the discussion section of the paper (page 9) to address the possible differences in the data from the Eaton paper versus our data. Eaton et al. used a different Gal4 driver, Mhc-Gal4 which is expressed later in development than the Mef2-Gal4 driver we used. We did test the MhcGal4 driver and found it to be ineffective at depleting dynein by RNAi. Our experiments also used RNAi depletion rather than expression of a dominant negative P150Glued construct. The dominant negative construct may not inhibit all functions of the endogenous dynein/dynactin complex to the same extent as RNAi depletion.

In addition, similar to our results, the Eaton paper reported no effect on the Dlg but also said there was no effect on Glutamate receptors. While they show the Dlg images, they do not show the Glutamate receptor clusters and may not have been looking for an increase in receptor field size. Depending on the magnification used, this may not be strikingly obvious.

Minor points:
1. Please explain how 'HRP' labels neurons (nothing is included in the methods). Is this short-hand for anti-HRP labelling? If so, include appropriate citations for why this works.

This is a standard method for visualizing the presynaptic side of the NMJ in Drosophila. As requested, we have included in the methods section (page 13) the citation from the Jan Lab that reported the neuronal specificity of horseradish peroxidase (Jan LY, Jan YN. PNAS 1982. PMID 6806816).

2. Please explain for the non-expert how different synapse types are identified in this system.

As requested, a citation on a book chapter from the book The Drosophila Neuromuscular Junction: Structure and Function, that explains the differences has been cited in the text on page 3, (Gramates and Budnik, 1999).

3. Does depletion of khc lead to a more disorganized dhc pattern (Fig. 1F)?
We do not observe any obvious pattern change around the NMJ. There is also a lot of tracheae in the image referred to. Tracheae also stains positive for dynein.

4. Figure S2A contains data from Fig. 2. I would suggest putting all the data in fig. 2. The significance of the elavGal80 should be discussed at this point (p4), rather than later (p5).

As suggested, we have moved this data from figure S2A to Figure 2 and mentioned the use of the elavGal80 in the text earlier.

© 2025. Published by The Company of Biologists under the terms of the Creative Commons Attribution License (https://creativecommons.org/licenses/by/4.0/).

5. The method for analysing GluRII distribution is hard to understand. How reliable is the method, which seems to calculate spheres, whereas the label is clearly in patches? Perhaps a worked example in supplemental material would help. In the analysis of BRP puncta distribution, does the total number of BRP spots change, or their intensity? How is a GluRII spot defined in these images? Many look like stripes.

It is not really calculating "spheres" but finding distinct patches of GluRII staining that are not touching. These analyses are done in 3D. The scripts are available on GitHub and described in the methods section (pages 14-15). The volume of the spheres measured were used to estimate a spherical radius. A radius measurement was used for comparison to previous analysis methods that used the diameter of a sphere (Pielage et al., 2006-PMID: 17088429).

We did not do intensity measures of BRP puncta and it is unclear what changes in intensity would mean if they were present.

We looked at the total number of BRP puncta per NMJ, and there is not a significant difference. As requested, this is now included as Figure S2K.

6. All the figures include red-green merges, although b+W images are also provided. The authors should consider using green and magenta for the benefit of colour-blind readers.

As requested, we have changed the figures to magenta and green.

7. The manuscript would benefit from some changes to the writing style. The present tense is used too much to describe results.
There is also repetition of results text in the figure legends, and little description of the actual experimental detail.

There was a stylistic preference to write the results in the present tense.

8. The figure legends provide numbers of NMJs analysed, and the methods state the number of animals used, but it would be better to have all of that information in the legends (e.g. 20 NMJs from 6 animals).
As requested, we have included the requested information in the figure legends.

*****

Reviewer 2 Comments for the Author:
1: The authors analyzed the distribution of acMT at the NMJ. They faintly overlap with b-spectrin. What is the distribution of non-acetylated MTs at NMJs.

As requested, we now show the distribution of all microtubules and the distribution of tyrosinated microtubules (Figure 1 and S1).

2: The description concerning the figure 3 E-G in the result section is difficult to follow. It is not clear from the text that bruchpilot is located on the presynaptic side. It is not clear neither what we learn by following the brp/receptor ratio . From the Sim analysis, in control, brp and GluRIIC partially overlap . In absence of dynein, brp appears inside the cluster but excluded from GluRIIC signal.

We clarified in the text what the ratio means on the top of page 5. Essentially, it is another indicator that the sizes of the clusters are larger and the distribution of presynaptic components per cluster is disrupted which can lead to the physiological differences we observe. The SIM images are shown in 3D reconstruction SUM images. In both cases, the GluRIIC signal is absent from the BRP signal in single section images.

Reviewer 3 Comments for the Author:
General criticism:
The authors convincingly demonstrate that a knock down of postsynaptic Dynein severely impairs the cytoskeletal organization of the postsynaptic SSR. The observed increase in

© 2025. Published by The Company of Biologists under the terms of the Creative Commons Attribution License (https://creativecommons.org/licenses/by/4.0/).

GluR cluster size and the corresponding increase in miniature amplitudes would be entirely consistent with the previously reported requirement of postsynaptic beta-spectrin for these processes (Pielage et al., 2006). In principle, also their model that PIP2 (potentially generated by Skittles at the postsynaptic membrane) regulates beta-spectrin complex assembly is supported by the nice demonstration of a direct interaction between PIP2 and Hts published in Wang et al., 2014.
However, as the authors only correlate the phenotypes of different knock down situations that all result in very variable effects of potential target genes it is currently not possible to deduce meaningful mechanistic insights from this work. The authors should complement their approaches with targeted point mutations or rescues (or at least overexpression) of mutant isoforms lacking interaction domains to determine cause and consequence.
Currently, very strong conclusions are made that are not supported and the authors fail to address alternative possibilities that could equally explain the data. As an example, the impairment of postsynaptic beta-spectrin localization may be responsible for the observed reduction in Hts level. Despite the potential "upstream" position of the adaptor protein Ankyrin as an interactor of CAMs, its postsynaptic localization entirely depends on beta-spectrin.
Similarly, it is not consistent that e.g., Dlg level or distribution are not at all affected in the Dhc knock down, if beta-spectrin and Hts levels are indeed reduced to less than 30%. It has been reported that loss of Hts and beta-spectrin impair Dlg level (Hts) and localization (beta-spectrin) (again, addressed in Pielage et al., 2006 and Wang et al., 2014).

With all due respect to the reviewer, there appears to be a difference of opinion on the interpretation of experiments reported in the publications cited by the reviewer. In our view, some of the reviewer's statements are an over interpretation or an oversimplification of the published data.

To the reviewers point on Hts, complete loss of Hts in a null mutant background results in a reduction in Dlg (Wang 2011), but this reduction was only 30%. We still see Hts/Adducin present by antibody staining when Dynein is depleted, so it is unexpected that this would give the null Hts phenotype (a 30% reduction in Dlg). Additionally, it was unclear, in the Wang et al paper, if this phenotypic reduction in Dlg was due to an effect on SSR formation or postsynaptic loss of Dlg, as overexpression of Hts results in a more substantial loss of Dlg (Wang 2011). As it appears to be phosphorylation levels of Hts that mediate the interaction with Dlg (Wang 2014), we cannot make any assumptions about the levels of phospho-Hts that are present and what the expectation on Dlg localization changes would be.

In the Pielage 2006 paper, Loss of B-Spectrin results in a disorganization of Dlg. levels of Dlg were not quantified nor do they appear to be substantially changed in levels. We noted in these experiments that B-Spectrin and alpha spectrin was lost throughout the muscle, not just at the postsynaptic membrane. We only observe loss of B-Spectrin at the postsynaptic membrane, not throughout the muscle, when dynein is depleted. We do not observe disorganized Dlg like the Pielage 2006 paper, but this paper also points out that there is a broadening of the SSR, where Dlg localizes. The loss of B-spectrin through the muscle likely disrupts more than just the postsynaptic membrane organization and this loss may explain the disorganization of Dlg that the Pielage paper observed. Of note, we have looked at Dynein localization at the postsynaptic membrane when B-Spectrin is depleted. We found that Dynein is significantly reduced upon B-Spectrin depletion (Figure S6E), suggesting that B-Spectrin loss throughout the muscle impacts the microtubule cytoskeleton and trafficking. <u>We will now include this data in the manuscript Figure S6 and our observations that the microtubule cytoskeleton appears to be disorganized as well.</u>

<u>We have included the possible interpretation that dynein could impact both Skittles/PIP2 and separately B-Spectrin (page 8). However, we address in the discussion (page 11) why we believe that B-Spectrin reduction is not the major contributor to the GluR enlarge receptor fields we observe when dynein is depleted</u>. First, we observe similar levels of knockdown of B-Spectrin in the dynein depletion background and in the Skittles depletion background, however Skittles depletion only has a negligible effect on glutamate receptor size. Second, our results, as described above, suggest that B- spectrin is needed throughout the muscle membrane to aid in microtubule organization and dynein localization at to the postsynaptic membrane. Lastly, other groups have

© 2025. Published by The Company of Biologists under the terms of the Creative Commons Attribution License (https://creativecommons.org/licenses/by/4.0/).

also shown a loss of the postsynaptic spectrin cytoskeleton that does not impact glutamate receptor organization (Christophers et al, 2024; Restrepo et al, 2022; Wang et al, 2018).

To attempt to specifically address the roles of postsynaptic Β-Spectrin and Skittles, we tried to target Β-Spectrin and Skittles specifically to the postsynaptic NMJ in dynein depleted muscles, however the constructs used (MHC-CD8-Sh-BSpec and MHC-CD8- Sh-Sktl) did not target as expected so we could not interpret the result.

We are unsure why the review brought up Ankyrin as we did not look at Ankyrin.

Loss of Dynein clearly disrupts postsynaptic organization but the mechanistic reasons for this are still unclear, and the claims of the authors are currently insufficiently supported.

Specific points:
1) The authors claim that the localization of Dynein to the postsynaptic SSR depends on its motor function and thus on a MT based transport. However, while the authors show in Figure 1 that tubulin is present in the vicinity of postsynaptic SSR (marked by b-spectrin), the MT organization does not support a potential targeting of Dynein or Dynein-cargo to GluR clusters. Currently only maximum projections of 3D image stacks are shown that do not allow a 3D reconstruction of MT localization. The highlighted MTs may be below the synaptic bouton. Single sections or a 3D SIM reconstruction would be necessary to demonstrate that MTs reach GluR clusters to support the proposed MT-dependent Dynein localization at the GluRs that would be necessary for e.g., the transport of Skittles.

The reviewer may have missed supplemental Table 1. Here we clearly state that image 1A is a single section, not a max projection or a Sum image. This is not a 3D reconstruction, but rather a single section. We have added the fact that this is a single section to the figure legend as well. The point of this figure was to show that microtubules are not completely excluded from the SSR.

2) Similarly, the claim that Dynein localizes to the center of GluR clusters should be supported by 3D reconstructions of the SIM images. In addition, the data should be quantified. From the images presented in Figure 3A`` and Figure 8G´, H´ only very few Dynein punctae localize to GluR clusters - others are at a clear distance or next to GluR clusters. 3D data and quantifications are necessary to support this claim.

The images in Figure 8G-H' (Now 7D-E) are SIM 3D reconstructions. 3A is a single section so the expectation would not be complete overlap because of the sectioning, The claim is not that every Dynein punctae is associated with every glutamate receptor. As stated in the text (page 4, paragraph 4) "the distribution of dynein aligns with the pattern of glutamate receptors (Figure 3A-A''), with some overlap (white in inset 3A'')", and a central localization pattern in SIM images (Figure 7). **The observation that dynein localizes to receptor clusters, is a new observation and provocative.**

Do the authors observe a similar localization for Dlic-GFP? – This is not clear from Figure S1.

The dynein antibody P1H4 is a specific monoclonal antibody (McGrail et al., 1997). Figure S1 does not report on both Dlic-GFP and Glutamate receptor clusters, only the localization of Dlic-GFP. Moreover, in this wildtype background, endogenous Dlic would compete for Dlic-GFP localization to the glutamate receptor clusters.

4) All GluR phenotypes are consistent with the absence of beta-spectrin. However, the absence does not seem to be 74% at all NMJs. Such a decrease should result in a severe disruption of Dlg (see above). In Figure 5D still significant levels of beta-spectrin are evident at the NMJ in the background (partially below the inset). The authors should plot individual data points to allow a better judgement of variability (this applies to all data sets presented)

Again, it seems the reviewer has a strong expectation of what the results should be, based on a previous set of experiments. We are just reporting our data. Not only where the experiments

© 2025. Published by The Company of Biologists under the terms of the Creative Commons Attribution License (https://creativecommons.org/licenses/by/4.0/).

reference conducted differently (see next paragraph below), but in Pielage 2006, there is no measurement of Dlg depletion, only evidence for the disruption of localization. Moreover, in Wang et al (2011) an Hts null background reduces Dlg levels by 30%. In our case, our measurement of Hts levels after dynein depletion is still ~70% and there is no change in Dlg.

The Beta-Spectrin network is found throughout the muscle, not just at the postsynaptic domain, therefore loss of Beta-Spectrin would be expected to have severe effects on multiple proteins. In our observations, when dynein is depleted B-Spectrin is only affected at the postsynaptic membrane. The B-Spectrin network throughout the muscle tissue in our images is intact (Figure 5B). Further, when B-Spectrin is depleted from the muscle, we show that postsynaptic dynein localization is disrupted (Figure S6E). This suggests that B-Spectrin depletion may independently affect postsynaptic glutamate receptor localization through disruption of dynein. We have now included this data in our revision.

[Note: The variability in the data sets are indicated by the error bars.]

5) The authors propose a linear sequence from Skittles via PIP2-Hts to beta- spectrin. Why are PIP2 and Hts level much less affected than beta-spectrin levels in the absence of Dhc64c? Can the small reductions in PIP2 level indeed explain the dramatic loss of beta-spectrin?

To address the reviewer's point, we have modified the text to include the alternative possibility that dynein could independently affect B-Spectrin levels (page 8).

The antibody signals and the PIP2 reporter are not perfect readouts of the protein levels, as different antibodies have different affinities and avidities for the protein they are targeting and there can also be different levels of background signal. Thus, comparing the B-Spectrin signal level to the Hts levels or the PIP2 levels isn't a fair comparison, particularly in the case where PIP2 is being recognized by a GFP-reporter that binds to PIP2. We suggest that the comparison can only be made between the controls and dynein depletion (or other protein depletion) for each antibody separately.

6) The authors claim on page 8: "Together these results suggest that dynein is required to transport Skittles to the NMJ and that Skittles localization is required to locally produce postsynaptic PIP2." However, they do not provide any data supporting these claims. Why do the authors omit an analysis of PIP2 abundance in Skittles RNAi or overexpression animals? Does it indeed locally regulate PIP2?

As requested, we have done this experiment, and as expected PIP2 levels are diminished when Skittles is depleted (now in Figure S5A).

What happens to Hts localization after Skittles knock down? Do levels go down in correlation with a reduction in PIP2 and does this sufficiently explains the observed reduction in beta-spectrin?

As above, we cannot directly compare different antibody signal levels. Looking at Hts goes beyond the scope here, nor do we see that we would really gain any more information by doing this experiment.

The image in Fig. 7 J still shows much higher beta-spectrin level than observed after the knock down of Dhc64c and not like a 62% knock down. Again, the level of beta-spectrin seem to directly correlate with GluR clustering.

We tried to show representative images. The quantifications were all done in the same way and from these quantifications we saw a 62% reduction in B-Spectrin compared to the control. The reviewer seems to be over interpreting what they "feel" they are seeing in the image as compared to quantification of the data. In the text, we have included the alternative possibility (page 8) that Dynein also independently affects B-Spectrin which may in turn influence glutamate receptors. However, our data also suggests that the spectrin cytoskeleton in the muscle affects microtubule organization and dynein localization at the NMJ (Figure S6A-E).

© 2025. Published by The Company of Biologists under the terms of the Creative Commons Attribution License (https://creativecommons.org/licenses/by/4.0/).

7) The observed effects on GluRIIC levels in the Skittles are not supported by the data. In the SIM analysis in Fig. 8E there does not seem to be an effect on either GluRIIC abundance or changes in membrane/ extrasynaptic GluRIIC levels. The reported defect in Fig. 8 B, C is simply not evident in the higher quality SIM analysis.

SIM images are widefield images and are very different from confocal images. With respect to “higher quality” images, this is not necessarily the case, rather SIM provides better spatial resolution than we can get with confocal imaging. It is hard to tell if these punctae in the SIM images are antibody background versus real puncta, we will remove the interpretation of a decrease in puncta throughout the muscle as we have no way of determining if these are antibody background or true GluRII receptor containing punctae.

8) The authors cannot claim that Dynein acts directly to position GluR clusters only on co-localization data - how is this supposed to work. Does it bind directly to GluRs - this should then be demonstrated biochemically.

We agree that we have not definitive demonstrated a mechanism for how dynein would position GluR clusters. Nonetheless, this is the first observation that dynein function is somehow involved in the organization of GluR receptor clusters. We believe this will be of interest to the readers. Based on our data, we can speculate about a mechanism here, but determining the exact mechanism will require additional studies.

Minor points
1) The authors demonstrate that GluRII cluster size is increased in the absence of Dhc64c. The current blotting of the data shows the size increase for GluRIIC clusters, but it is unclear whether the significant difference in GluRIIA and B radius is only due to very few larger clusters. A histogram of all data points may facilitate data interpretation.

Because of the high background staining with the GluRIIA and GluRIIB antibodies, we also used the cleaner MHC-Myc-GluRIIA and MHC-Myc-GluRIIB genetic expression constructs and a commercially available Myc antibody signals to confirm the observation that receptor cluster size is enlarged. The quantification confirming the observation is provided in the Box and Whisker plots (Figure S3D). We have moved the GluRIIA and GluRIIB endogenous staining analyses to figure S2B. Box and Whisker plots indicate a significant difference in the average size of the receptor cluster.

<u>Second decision letter</u>

MS ID#: jcs.263844

MS Title: Dynein-Driven Regulation of Postsynaptic Membrane Architecture and Synaptic Function

Authors: Amanda L. Neisch; Thomas S. Hays; Adam W. Avery; Mingang Li; Thomas Pengo

I am happy to tell you that your manuscript has been accepted for publication in Journal of Cell Science, pending standard publication integrity checks.

© 2025. Published by The Company of Biologists under the terms of the Creative Commons Attribution License (https://creativecommons.org/licenses/by/4.0/).
